# Oxidative Damage and Antioxidant Response in Frontal Cortex of Demented and Nondemented Individuals with Alzheimer's Neuropathology

**DOI:** 10.1523/JNEUROSCI.0295-20.2020

**Published:** 2021-01-20

**Authors:** Anna Fracassi, Michela Marcatti, Olga Zolochevska, Natalie Tabor, Randall Woltjer, Sandra Moreno, Giulio Taglialatela

**Affiliations:** ^1^Mitchell Center for Neurodegenerative Diseases, Department of Neurology, University of Texas Medical Branch (UTMB), Galveston, Texas 77550; ^2^Neuroscience Summer Undergraduate Program, University of Texas Medical Branch, Galveston, Texas 77555; ^3^Department of Pathology, Oregon Health and Science University, Portland, Oregon 97239-3098; ^4^Department of Science, LIME, University Roma Tre, 00146 Rome, Italy

**Keywords:** Alzheimer's disease, miRNA-485, NDAN, oxidative stress, PGC1α, PPARα

## Abstract

Alzheimer's disease (AD) is characterized by progressive neurodegeneration in the cerebral cortex, histopathologically hallmarked by amyloid β (Aβ) extracellular plaques and intracellular neurofibrillary tangles, constituted by hyperphosphorylated tau protein. Correlation between these pathologic features and dementia has been challenged by the emergence of “nondemented with Alzheimer's neuropathology” (NDAN) individuals, cognitively intact despite displaying pathologic features of AD. The existence of these subjects suggests that some unknown mechanisms are triggered to resist Aβ-mediated detrimental events. Aβ accumulation affects mitochondrial redox balance, increasing oxidative stress status, which in turn is proposed as a primary culprit in AD pathogenesis. To clarify the relationship linking Aβ, oxidative stress, and cognitive impairment, we performed a comparative study on AD, NDAN, and aged-matched human postmortem frontal cortices of either sex. We quantitatively analyzed immunofluorescence distribution of oxidative damage markers, and of SOD2 (superoxide dismutase 2), PGC1α [peroxisome proliferator-activated receptor (PPAR) γ-coactivator 1α], PPARα, and catalase as key factors in antioxidant response, as well as the expression of miRNA-485, as a PGC1α upstream regulator. Our results confirm dramatic redox imbalance, associated with impaired antioxidant defenses in AD brain. By contrast, NDAN individuals display low oxidative damage, which is associated with high levels of scavenging systems, possibly resulting from a lack of PGC1α miRNA-485-related inhibition. Comparative analyses in neurons and astrocytes further highlighted cell-specific mechanisms to counteract redox imbalance. Overall, our data emphasize the importance of transcriptional and post-transcriptional regulation of antioxidant response in AD. This suggests that an efficient PGC1α-dependent “safety mechanism” may prevent Aβ-mediated oxidative stress, supporting neuroprotective therapies aimed at ameliorating defects in antioxidant response pathways in AD patients.

## Significance Statement

The present study importantly contributes to clarifying the molecular events underlying age-related AD pathology, emphasizing the role of antioxidant defenses against Aβ toxicity. Specifically, we addressed the mechanisms whereby a particular group of individuals, referred to as nondemented with AD neuropathology, resists dementia, despite displaying amyloid and tau pathology consistent with fully symptomatic AD. This study reveals the ability of these individuals to activate an efficient antioxidant response to cope with oxidative stress, possibly representing one of the mechanisms by which they remain cognitively intact. Our work, in addition to advancing the knowledge on the role of oxidative stress in AD, may lay the foundation for novel therapeutic approaches to the disease, possibly based on activation of the peroxisome proliferator-activated receptor γ-coactivator 1α-mediated antioxidant pathway.

## Introduction

Alzheimer's disease (AD) is a progressive neurodegenerative disorder, histopathologically characterized by extracellular amyloid β (Aβ) plaques and intracellular neurofibrillary tangles (Querfurth and LaFerla, 2010; DeTure and Dickson, 2019). Several mechanisms have been proposed to explain AD pathogenesis, among which is the so-called amyloid cascade, involving a critical role of Aβ peptide (Hardy and Higgins, 1992; Selkoe and Hardy, 2016). However, the correlation between Aβ accumulation and dementia (Holtzman et al., 2011; Musiek and Holtzman, 2015) has been challenged by the emergence of a group of individuals recently classified as A^+^T^+^N^–^ (Jack et al., 2018), and here referred to as “nondemented with Alzheimer's neuropathology” (NDAN). Despite harboring neuropathological features of AD (Bjorklund et al., 2012), they remain cognitively intact (Zolochevska and Taglialatela, 2016). The existence of NDAN suggests that some unknown mechanisms are triggered to resist the detrimental events that otherwise lead to cognitive impairment in AD. Such mechanisms, while not impeding Aβ overproduction or aggregation, possibly prevent neurotoxic effects of the peptide. Noteworthy, Aβ accumulation affects mitochondrial redox balance increasing oxidative stress, which has been proposed to be a primary culprit in AD pathogenesis (Mecocci et al., 1994; Hensley et al., 1995; Markesbery, 1997; Smith et al., 2000; Butterfield et al., 2001; Lauderback et al., 2001; Sultana and Butterfield, 2013; Zhao and Zhao, 2013; Bonda et al., 2014; Wang et al., 2014; Kim et al., 2015; Luca et al., 2015; Huang et al., 2016; Sanabria-Castro et al., 2017; Cheignon et al., 2018). Based on this evidence, we hypothesize that the resistance to dementia in NDAN patients could be related to their ability to cope with reactive oxygen species (ROS) overproduction, by activating an efficient antioxidant response.

Redox imbalance triggers an array of cellular mechanisms, including activation of transcription factors, that regulate energy metabolism and antioxidant defenses (Clark and Simon, 2009). Among these, peroxisome proliferator-activated receptor (PPAR) γ-coactivator 1α (PGC1α) regulates genes involved in glucose and lipid metabolism, mitochondrial biogenesis, and antioxidant response (Bagattin et al., 2010; Katsouri et al., 2012; Wenz, 2013). PGC1α also coactivates the PPARα isotype, a major regulator of peroxisomal and mitochondrial biogenesis and functions (Feige et al., 2006; Wenz, 2011). PPARα is known to be modulated in neurologic disease, including AD (Fanelli et al., 2013; Porcellotti et al., 2015), and several studies emphasize the possible treatment of AD based on PPARα natural or synthetic ligands (Santos et al., 2005; Inestrosa et al., 2013; Fidaleo et al., 2014; D'Orio et al., 2018).

Our previous *in vivo* investigations (Cimini et al., 2009; Fanelli et al., 2013; Porcellotti et al., 2015), conducted in the Tg2576 mouse model of AD (Hsiao et al., 1996), showed significant variations of antioxidant enzymes expression levels and ensuing oxidative damage at the onset and during the progression of disease. These changes were accompanied by altered expression of both PPARα and PGC1α in mouse hippocampus and neocortex, starting from 3 months of age.

To transfer these observations to the human AD brain and clarify the relationship linking Aβ, oxidative stress, and cognitive impairment, we performed a comparative study on AD, NDAN, and normally aged human postmortem frontal cortices, focusing on possible differences concerning antioxidant response mechanisms against oxidative stress. To assess the precise cellular localization of oxidative damage, we evaluated the occurrence of 8-oxo-dG marker and 4-hydroxy-2-nonenal in neuronal and astroglial cells, by quantitative double immunofluorescence (IF). To gain information about the specific antioxidant capacity of neurons and astrocytes in AD and NDAN individuals, we studied the expression and distribution of superoxide dismutase 2 (SOD2). Considering the role of PGC1α and PPARα as redox sensors and regulators of SOD2 transcription, we investigated the localization of these factors in neurons and astrocytes. Furthermore, given the central role of peroxisomes in ROS metabolism (Schrader and Fahimi, 2006; Pascual-Ahuir et al., 2017), we investigated the expression and localization of catalase (CAT), whose levels are regulated by PPARs and PGC1α (St-Pierre et al., 2006; Shin et al., 2016).

Understanding protective molecular and cellular processes underlying NDAN ability to resist Aβ-mediated detrimental effects should be of help in revealing novel targets for the development of effective therapeutic approaches for AD.

## Materials and Methods

### 

#### 

##### Human subjects and autopsy of brain tissues

Postmortem brain tissues were obtained from the Oregon Brain Bank at Oregon Health and Science University (OHSU; Portland, OR). Donor subjects of either sex were enrolled and clinically evaluated in studies at the National Institutes of Health-sponsored C. Rex and Ruth H. Layton Aging and Alzheimer's Disease Center (ADC) at OHSU, in accordance with protocols that were approved by the OHSU Institutional Review Board (IRB). Informed consent was obtained from all participants before their enrolment in the studies at the ADC. Subjects were participants in brain-aging studies at the ADC and received annual neurologic and neuropsychological evaluations, with a clinical dementia rating (CDR) assigned by an experienced clinician. A neuropathological assessment was performed at autopsy, and in compliance with IRB-approved protocols. A neuropathologist scored autopsy brain tissue for Aβ plaques and neurofibrillary tangles, according to standardized CERAD (Consortium to Establish a Registry for Alzheimer's Disease) criteria and Braak staging. Participants were classified as having AD when possessing a National Institute for Neurologic and Communicative Disorders and Stroke–Alzheimer's Disease and Related Disorder Association diagnostic criteria for clinical AD (CDR) including a Mini-Mental State Examination (MMSE) score <10. Control (ctrl) participants performed normally in cognitive examinations (MMSE score, 29–30). NDAN case patients displayed little to no cognitive impairment (MMSE score, ≥27), though were found at autopsy to have amyloid plaques and neurofibrillary tangles comparable to fully symptomatic AD (Table 1). Donor subject samples were deidentified by ADC before being provided to University of Texas Medical Branch (UTMB), so that no approval was required from the UTMB IRB under CFR §46.101(a)(1). The cases used in this study are described in Table 1.

**Table 1:** Clinical data of the subjects used in the study

Case no.	Diagnosis	Age (years)	Sex	Braak stage	MMSE	PMI (h)
767	Ctrl	86	F	2	29	8
785	Ctrl	83	M	1	29	<14
1104	Ctrl	86	F	2	29	16
1229	Ctrl	>89	F	2	30	12
1525	Ctrl	89	F	1	29	3
1731	Ctrl	74	F	2	29	7.5
2467	Ctrl	99	F	3	28	4.5
2553	Ctrl	100	M	2	28	4
2682	Ctrl	90	F	2	29	9
2755	Ctrl	95	F	2	29	18
2953	Ctrl	100	F	3	27	2.5
3200	Ctrl	90	M	2	20	4.5
1538	AD	84	M	5	6	5.5
1678	AD	76	F	6	1	25
1688	AD	75	M	6	0	17
1774	AD	>89	M	6	2	3.25
1776	AD	>89	F	6	6	6.25
1777	AD	67	F	6	9	20.5
2312	AD	87	F	6	NA	2.5
2315	AD	95	M	4	NA	4
2317	AD	88	M	6	NA	4
2318	AD	74	F	6	NA	2
697	NDAN	>89	M	5	29	5
1016	NDAN	>89	F	6	26	8
1095	NDAN	87.8	M	4	29	3
1179	NDAN	>89	F	4	27	2.5
1362	NDAN	>89	F	4	27	48
1578	NDAN	89	M	5	27	15.5
1686	NDAN	87	F	4	29	2.5
1845	NDAN	86	M	4	29	4.5
2376	NDAN	93	M	4	26	4
2474	NDAN	90	F	4	28	8
2980	NDAN	98	F	4	27	4
3178	NDAN	93	M	3	29	10

Braak stage, A measure of the number and location of tau tangles and Aβ plaques in the brain; MMSE, Mini Mental State Examination (administered within the last year); PMI, Postmortem interval; F, female; M, male. Average PMI: ctrl, 8.09 h; AD, 9 h); NDAN, 9.68 h.

**Figure 1.**
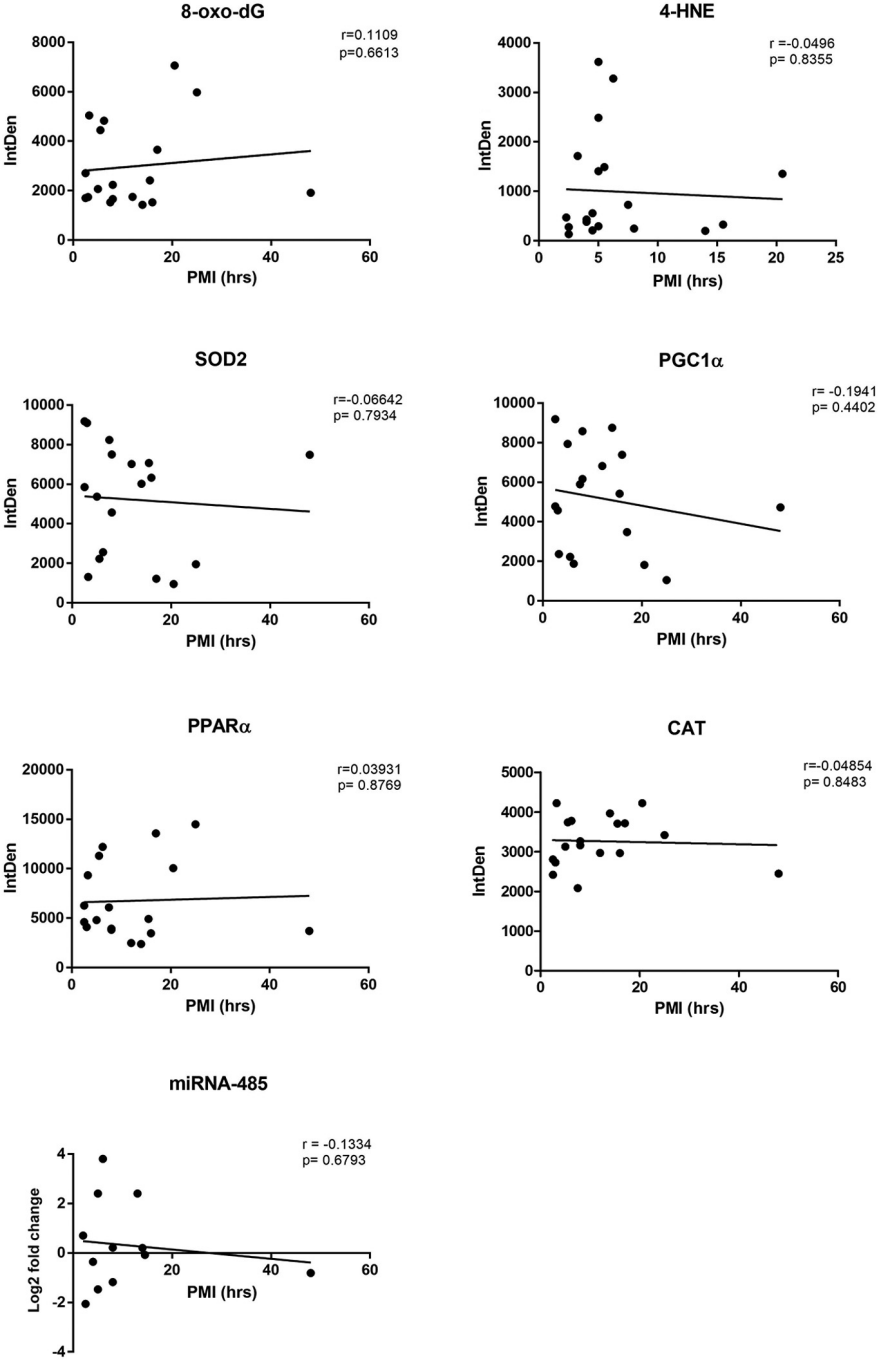
Correlation analysis between each of the studied parameters and PMI values across all of the assayed specimens. A Pearson's correlation test was performed for each measurement against the PMI. Correlation coefficient (*r*) and *p* values are noted in the individual plots showing no significant correlation with PMI values.

To ensure that the variations in postmortem interval (PMI) did not affect any measurements, a correlation analysis between PMI values and results obtained in the various assays presented here was performed using a Pearson's correlation test. No correlation was found between PMI values and any of the elements/antigens assayed here (Fig. 1), and therefore observed differences could not be attributed to differences in nonspecific postmortem tissue degradation. However, although the results shown in Figure 1 reinforce the validity of the data regarding the different antigens studied here, it is nonetheless important to appreciate that brains obtained >10 h PMI might not necessarily fully reflect freshly obtained brain tissue.

##### Tissue processing and immunofluorescence

Fresh frozen cortical tissue blocks (*n* = 6/group) were removed from storage at −80°C, equilibrated at −20°C, embedded in O.C.T. (optimal cutting temperature) compound (catalog #4583, Tissue-Tek), and 10 μm-thick sections were collected onto Superfrost Plus slides (catalog #12–550-15, Thermo Fisher Scientific). Prepared slides were stored at −80°C until use. Slides were fixed in 4% paraformaldehyde in 0.1 m PBS, pH 7.4 for 30 min at room temperature (RT). Nonspecific binding sites were blocked with 5% bovine serum albumin (catalog #A4503-100G, Sigma-Aldrich)/10% normal goat serum (NGS; Thermo Fisher Scientific) and sections were permeabilized with 0.5% Triton X-100/0.05% Tween-20 for 1 h at RT. Slides were incubated with the following primary antibodies, diluted in PBS containing 1.5% NGS/0.25% Triton X-100 overnight at 4°C: rabbit anti-PPARα (1:200; catalog #ab8934, Abcam; RRID:AB_306869); rabbit anti-PGC1α (1:200; catalog #ab54481, Abcam; RRID:AB_881987); rabbit anti-CAT (1:200; catalog #ab16731, Abcam; RRID:AB_302482); rabbit anti-SOD2 (1:200; catalog #GTX116093, GeneTex; RRID:AB_10624558; mouse anti-8oxo-dG (1:250; catalog #4354-MC-050, R&D Systems; RRID:AB_1857195); rabbit anti 4-HNE (1:200; catalog #ab46545, Abcam; RRID:AB_722490); rabbit anti-Aβ (1:200; catalog #ab201060; RRID:AB_2818982, Abcam); mouse anti-NeuN (1:200; catalog #MAB377, Millipore; RRID:AB_2298772); rabbit anti-NeuN (1:500; catalog #ABN78, Millipore; RRID:AB_10807945); and chicken anti-GFAP (1:500; GFAP, Aves Labs; RRID:AB_2313547). Slides were washed in PBS before incubation with the appropriate Alexa Fluor-conjugated secondary antibodies [goat anti-rabbit Alexa Fluor 488; 1:400; catalog #A-11008 (RRID:AB_143165); goat anti-mouse Alexa Fluor 594; 1:400; catalog #A-11 032 (RRID:AB_2534091); goat anti-mouse Alexa Fluor 488; 1:400; catalog #A-10 680 (RRID:AB_2534062); goat anti-chicken Alexa Fluor 594; 1:400; catalog #A-11 042 (RRID:AB_2534099); all from Thermo Fisher Scientific) in PBS containing 1.5% NGS/0.25% Triton X-100 for 1 h at RT. Finally, slides were washed in PBS, treated with 0.3% Sudan Black B (in 70% EtOH) for 10 min to block lipofuscin autofluorescence, washed again with deionized water, and coverslipped using Fluoromount-G-containing 4′,6′-diamidino-2-phenylindole dihydrochloride (DAPI; catalog #0100–20, SouthernBiotech) and sealed.

##### Quantitative microscopy

All immunoreacted sections were acquired with either a Nikon eclipse 80i (Nikon) or a Keyence BZ-X800 microscope, by using 20× and 60× immersion oil objectives. For each subject, four sections were analyzed and five images per section were captured. Quantitative analysis was performed using ImageJ software (NIH; https://imagej.nih.gov/ij), analyzing the intensity of fluorescence for each marker [Integrated Density (IntDens)], when the overall distribution was studied. When the colocalization of each marker with either NeuN or GFAP was addressed, the count of the positive cells for each marker and either NeuN- or GFAP-positive cells over the number of total cells, was made. Representative images were composed in an Adobe Photoshop CC2020 format.

##### Tissue processing and Western blot analyses

Fresh frozen cortical tissue blocks derived from control, AD, and NDAN subjects (*n* = 7/group) were removed from storage at −80°C and used for Western blotting (Wb) analyses. RIPA buffer (catalog #9806S, Cell Signaling Technology) with 1% protease and phosphatase cocktail inhibitors was used to lysate tissues and synaptosomes to obtain the total protein fraction and the synaptosomal fraction, respectively. The synaptosomes were isolated from the cortical tissues by using a method very well established in our laboratory (Franklin and Taglialatela, 2016; Comerota et al., 2017; Franklin et al., 2019). Briefly, we lysed the cortical tissues by using the SynPER lysis buffer (catalog #87793, Thermo Fisher Scientific) with 1% protease and phosphatase cocktail inhibitors. The brain homogenates were centrifuged at 1200 × *g* relative centrifugal field (RCF) for 10 min at 4°C. The supernatants (containing the synaptosomes) were collected and centrifuged at 15,000 × *g* RCF for 20 min at 4°C. The synaptosomal pellets were resuspended in HEPES-buffered Krebs-like buffer (143.3 mm NaCl, 4.75 mm KCl, 1.3 mm MgSO47H_2_O, 1.2 mm CaCl_2_, 20.1 mm HEPES, 0.1 mm NaH_2_PO_4_, and 10.3 mm d-glucose, pH 7.4). The cytosolic protein fraction was obtained by using the Nuclear/Cytosol Fractionation Kit (catalog #K266-100, BioVision) according to the manufacturer protocol. Briefly, the cortical tissues were homogenized in 1–2 ml of ice-cold PBS and centrifuged at 500 × *g* for 2–3 min at 4°C. After adding 0.2 ml of the CEB-A mix, the pellets were vortexed vigorously on the highest setting for 15 s to be fully resuspend. The suspensions were incubated on ice for 10 min, and, after adding 11 µl of ice-cold Cytosol Extraction Buffer-B, the samples were centrifuged for 5 min at maximal speed and immediately the supernatants (cytosolic fraction) were transferred in clean prechilled tubes. All the protein extracts prepared as above were quantified by using the Pierce BCA Protein Assay Kit (catalog #23225, Thermo Fisher Scientific) and subjected to SDS-PAGE. Specifically, the protein expression levels in the single individuals were analyzed by using 20 µg of protein extracts. Moreover, an equal amount of proteins extracted from each individual/group was pooled together to obtain a total of three pools (control, AD, and NDAN) and a range of 70–100 µg of proteins was used. Proteins were transferred to GE Healthcare Protran Nitrocellulose Transfer Membrane (catalog #10600001, Sigma-Aldrich) at 85 V at 4°C. Membranes were blocked using Odyssey blocking buffer (catalog #927–60 001, LI-COR) for 1 h at RT and probed overnight at 4°C with either of the following primary antibodies: rabbit anti-PPARα (1:1000; catalog #ab24509, Abcam; RRID:AB_448110); rabbit anti-PGC1α (1:1000; catalog #ab54481, Abcam; RRID:AB_881987); rabbit anti-CAT (1:1000; catalog #ab16731, Abcam; RRID:AB_302482); rabbit anti-SOD2 (1:1000; catalog #GTX116093, GeneTex; RRID:AB_10624558); mouse anti-synaptophysin antibody (SYN; 1:10,000, catalog #ab8049, Abcam; RRID:AB_2198854); and mouse anti-β-actin (ACTB; 1:50,000; catalog #A1978, Sigma-Aldrich; RRID:AB_476692). All of the primary antibodies were prepared in a solution of 1× TBST and Odyssey blocking buffer (1:1). Membranes were than washed three times with 1× TBST for 10 min each and incubated for 1 h with LI-COR secondary antibodies (1:10,000) in 1× TBST/Odyssey blocking buffer at RT. The membranes were again washed three times for 10 min each. Wb were imaged using the Odyssey Infrared Imaging System application software version 3.0.30 (LI-COR). The band densities were analyzed using ImageJ software, and normalized using the densities of the loading control obtained by reprobing the membranes either for ACTB or SYN for total/cytosolic and synaptosomal fractions, respectively. Representative images were composed in an Adobe Photoshop CC2020 format.

##### Quantitative RT-PCR of miRNAs

Total RNA was isolated using Life Technologies TRIzol Reagent (Thermo Fisher Scientific) from postmortem frozen human cortices of control, AD, and NDAN subjects (*n* = 4/group). Approximately 100 mg of tissue was placed in TRIzol and homogenized using the Polytron homogenizer (Thermo Fisher Scientific). Chloroform was then added and the samples were spun down at 12 000 rpm for 15 min at 4°C. The aqueous phase was transferred to a new tube containing isopropanol. The samples were centrifuged at 12,000 rpm for 10 min at 4°C. Pellet was washed with ice cold 80% ethanol and air dried. The samples were resuspended in 40 µl nuclease free water. The RNA concentration was measured using NanoDrop 2000c (Thermo Fisher Scientific).

Reverse transcription was performed using miScript II RT Kit (catalog #218160, Qiagen) according to the manufacturer protocol. Briefly, 0.5 µg of RNA was reverse transcribed in a 20 µl reaction volume containing 4 µl of 5× HiSpec buffer, 2 µl of 10× miScriptNucleics mix, and 2 µl of miScript Reverse Transcriptase. The mix was incubated at 37°C for 1 h, then at 95°C for 5 min and placed on ice. The reverse-transcribed miRNA mix was diluted with nuclease-free water to a final concentration of 3 ng/µl. Real-time PCR was performed to quantitate miRNA in control, AD, and NDAN. miScript SYBR Green PCR Kit (catalog #218073, Qiagen) was used according to the manufacturer protocol. Briefly, the reaction was performed in 25 µl final volume in each well containing 3 ng of reverse-transcribed miRNA, 1× SYBR Green, Has_miR-485-5p_1, or Hs_RNU6-2 miScript primers (Qiagen). The reaction was performed in Mastercycler EPGradient S (Eppendorf). The samples were incubated at 95°C for 15 min to activate the polymerase followed by 40 cycles of amplification, as follows: 94°C for 15 s, 55°C for 30 s, and 70°C for 30 s. Standard melting curve was performed at the end. The levels of miRNA-485 were normalized to U6 small nuclear RNA. The relative fold change in expression of target miRNAs was determined using the comparative cycle threshold method (2^-ΔΔCt^), and the obtained values were then log2 transformed.

##### Statistical analysis

Statistical analyses were performed using GraphPad Prism version 8.4.3 software. *t* Test, one-way ANOVA with Tukey's *post hoc* test, or two-way ANOVA with Sidak's multiple-comparison test were used to detect significant differences between groups. Data were then expressed as the mean ± SD, and for all statistical analyses *p* < 0.05 was considered as statistically significant.

## Results

### Oxidative damage and antioxidant response

#### 8-oxo-dG and 4-HNE distribution in neurons and astrocytes

**Figure 2.**
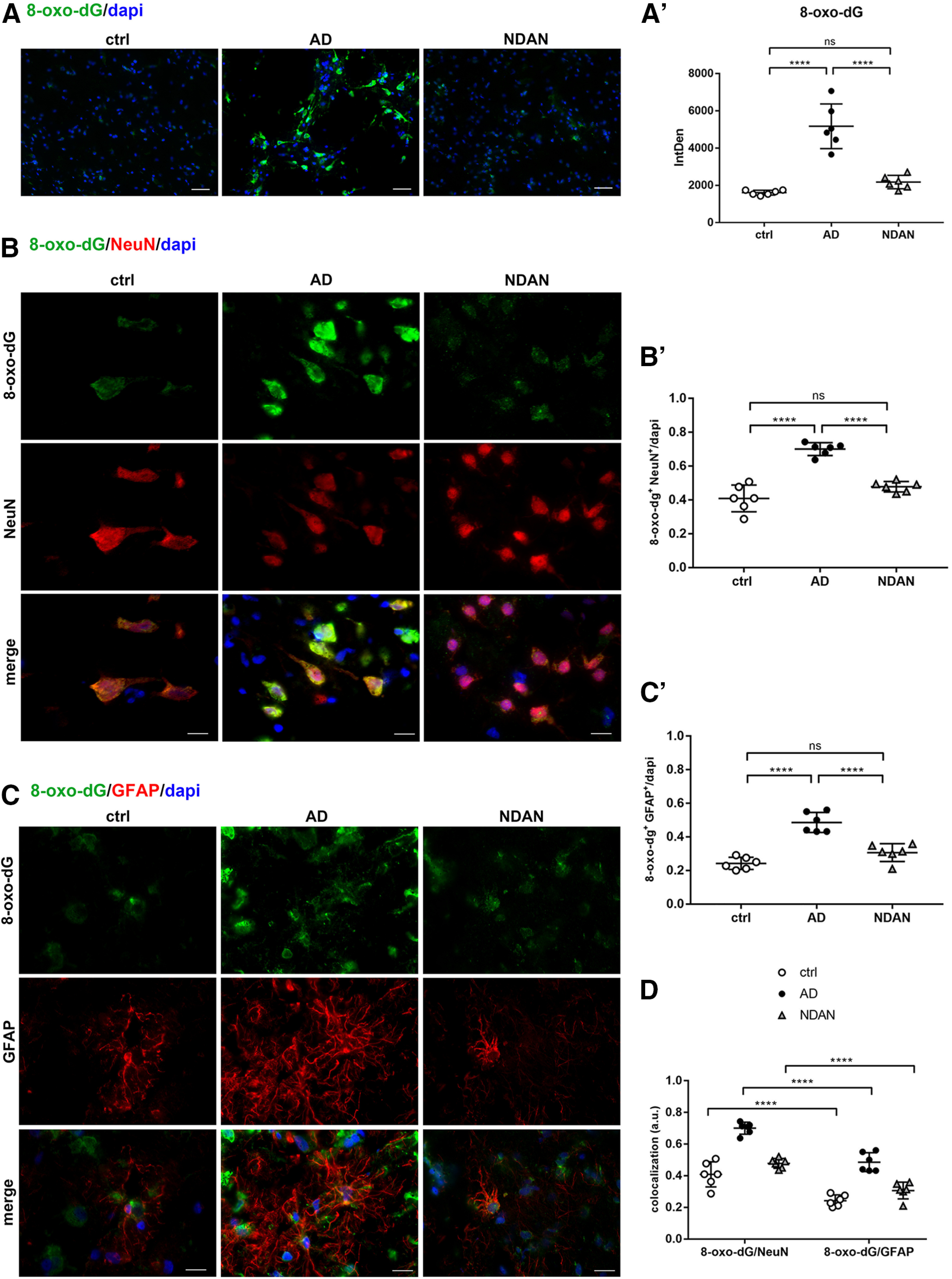
***A***, ***A′***, 8-oxo-dG expression and distribution in frontal cortex of control, AD, and NDAN subjects. Immunolocalization of 8-oxo-dG and quantitative analysis of IF images showing increased levels of oxidative damage in brains of AD subjects and low levels in NDAN subjects, compared with control subjects. Original magnification, 20×. Scale bar, 100 µm. Statistical analyses were made using one-way ANOVA (*F*_(2,15)_ = 41.67, *p* < 0.0001) following Tukey's multiple-comparisons test. Values are expressed as the mean ± SD. ****p < 0.0001. ***B–D***, 8-oxo-dG expression and distribution in frontal cortex neurons and astrocytes of control, AD, and NDAN subjects. ***B***, ***B′***, Double IF of 8-oxo-dG (green) in combination with NeuN (red) shows high levels of oxidative damage in AD neurons. NDAN neurons demonstrate low levels of oxidative damage marker. Magnification, 60×. Scale bar, 30 µm. The quantitative analysis of IF images shows significantly higher levels of oxidative damage markers in AD neurons. Statistical analyses were made using one-way ANOVA (*F*_(2,15)_ = 48.47, *p* < 0.0001) following Tukey's multiple-comparisons test. Values are expressed as the mean ± SD. ****p < 0.0001. ***C C′***, Double IF of 8-oxo-dG (green) in combination with GFAP (red) showing high levels of oxidative damage to astrocytes in AD subjects compared with control and NDAN subjects, although lower levels than in neurons. Magnification 60×. Scale bar, 30 µm. The quantitative analysis of IF images shows significantly higher levels of the oxidative damage marker in AD astrocytes, while NDAN and control astrocytes displayed comparable levels of damage. Statistical analyses were made using one-way ANOVA (*F*_(2,15)_ = 37.64, *p* < 0.0001) following Tukey's multiple-comparisons test. Values are expressed as the mean ± SD. ****p < 0.0001. ***D***, The analysis demonstrates relatively higher resistance of astrocytes to oxidative damage, compared with neurons, which appear more prone to AD-associated oxidative damage. Statistical analyses were made using two-way ANOVA (*F*_(2,30)_ = 80, *p* < 0.0001). Values are expressed as the mean ± SD. ****p < 0.0001. ns, not significant.

Considering the central role played by oxidative stress in AD pathogenesis and based on our previous data collected on the Tg2576 model (Fanelli et al., 2013; Porcellotti et al., 2015), we evaluated oxidative damage, occurring in the frontal cortex of AD, NDAN, and control subjects using 8-oxo-dG as a marker of oxidative DNA/RNA modifications. Interestingly, immunofluorescent staining predominantly localizes to the cytoplasmic compartment, indicating that such oxidative modifications selectively affect mitochondrial and cytosolic nucleic acids, rather than nuclear DNA (Fig. 2). When quantitatively evaluated by appropriate image analysis, 8-oxo-dG-immunoreactive levels appear significantly higher in AD versus control or NDAN individuals (ctrl vs AD, *p* < 0.0001; AD vs NDAN, *p* < 0.0001). These latter two brain samples indeed display consistently similar 8-oxo-dG immunoreactivity (ctrl vs NDAN, *p* = 0.3866; Fig. 2*A*′). To investigate the precise neural localization of the DNA/RNA damage, we performed double immunofluorescence of 8-oxo-dG in combination with NeuN—as a neuronal marker—or GFAP, an astroglial marker (Fig. 2*B–C*). In AD frontal cortices, both neurons (Fig. 2*B*,*B′*; ctrl vs AD, *p* < 0.0001; AD vs NDAN, *p* < 0.0001) and astrocytes (Fig. 2*C*,*C′*; ctrl vs AD, *p* < 0.0001; AD vs NDAN, *p* < 0.0001) display higher 8-oxo-dG immunoreactivity levels with respect to controls. Noteworthy, GFAP immunoreactivity appears especially intense in AD samples, revealing ongoing astrogliosis. By contrast, in NDAN frontal cortices, no astrogliosis was observed and 8-oxo-dG immunostaining was comparable to control, in either neurons (ctrl vs NDAN, *p* = 0.0991; Fig. 2*B*,*B′*) or astrocytes (ctrl vs NDAN, *p* = 0.1018; Fig. 2*C*,*C′*). Figure 2*D* summarizes the scenario in neurons and astrocytes in the three conditions highlighting that oxidative damage predominantly occurs in neurons, while astrocytes appear to be more resistant, showing a significantly fainter staining for 8-oxo-dG (ctrl vs ctrl, *p* < 0.0001; AD vs AD, *p* < 0.0001; NDAN vs NDAN, *p* < 0.0001).

To further confirm no effect of PMI length on the observed differences among groups, we evaluated the expression of 8-oxo-dG as a representative antigen among those presented here, also using brain samples from a different cohort with exceptionally short PMIs (Extended Data Fig. 2-1). The quantitative analysis showed significant differences among the three groups (ctrl vs AD, *p* = 0.0029; AD vs NDAN, *p* = 0.0019; ctrl vs NDAN, *p* = 0.9554; Extended Data Fig. 2-1*A*,*A′*) similar to what was observed in our primary case cohort and the Pearson's correlation test confirmed no correlation between PMI values and the variation of the expression of the antigen tested (Extended Data Fig. 2-1*B*).

10.1523/JNEUROSCI.0295-20.2020.f2-1 Figure 2-1 8-Oxo-dG expression and distribution in low-PMI brains. ***A***, ***A′***, 8-Oxo-dG immunostaining and quantitative analyses of control, AD, and NDAN frontal cortices (*n* = 4) with low PMIs (2–5 h) showing increased oxidative damage in AD subjects compared with control subjects and no significant differences between control and NDAN subjects. Original magnification, 60×. Scale bar, 30 µm. Statistical analyses were made using one-way ANOVA (*F*_(2,9)_ = 15.71, *p* = 0.0012), following Tukey's multiple-comparisons test. Values are expressed as the mean ± SD. **p < 0.01. ***B***, A Pearson's correlation test was performed for each measurement against the PMI. Correlation coefficient (*r*) and *p* values are noted in the individual plots showing no significant correlation with PMI values. Download Figure 2-1, TIF file.

**Figure 3.**
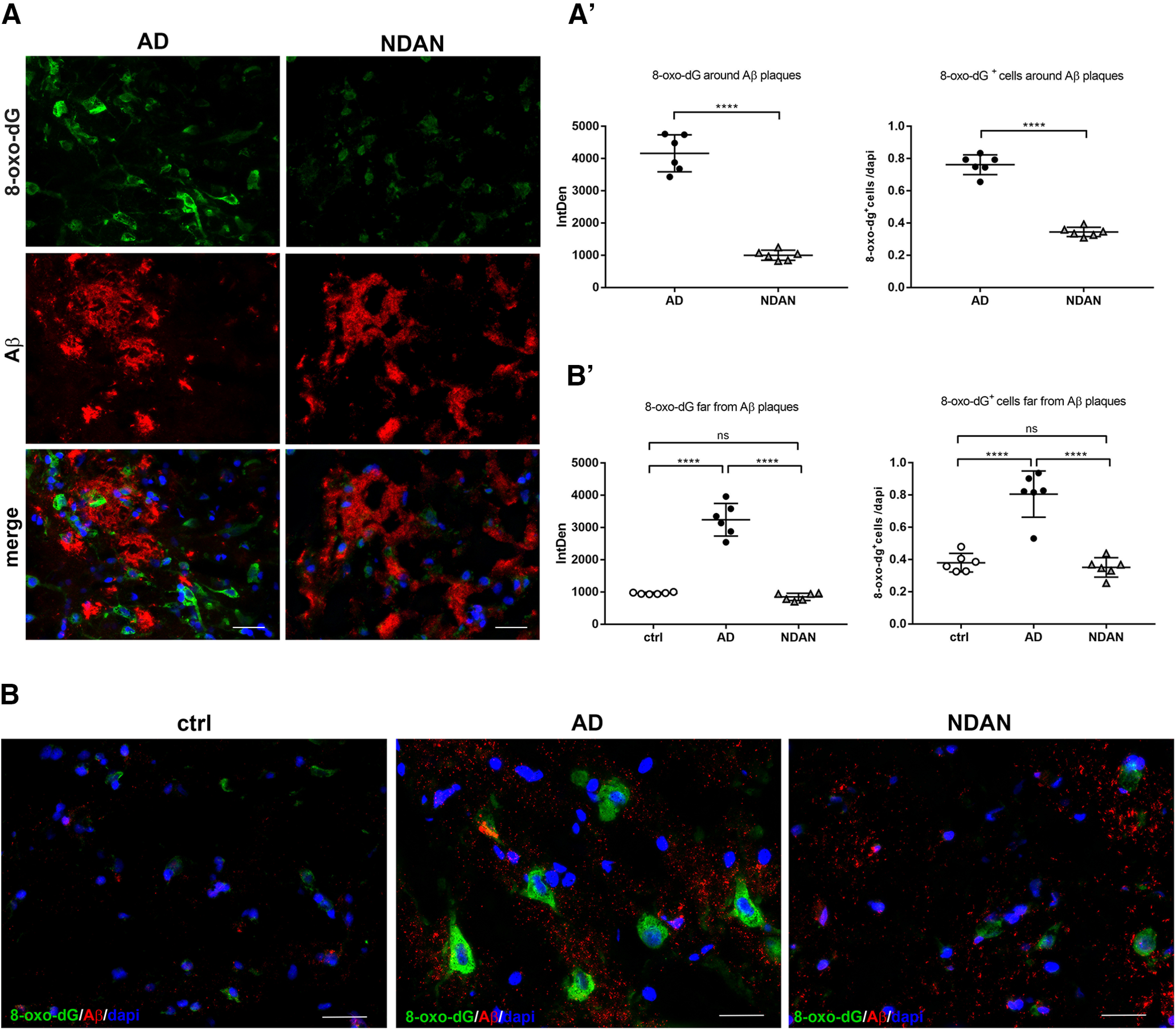
8-oxo-dG expression and distribution in relation to Aβ accumulation. ***A***, ***A****′*, Double IF of 8-oxo-dG (green) and Aβ (red) showing the oxidative damage to nucleic acids around Aβ plaques in AD and NDAN subjects. The quantitative analyses in terms of the intensity of fluorescence (*t*_(10)_ = 13.06, *p* < 0.0001, unpaired *t* test) and number of 8-oxo-dG^+^ cells (*t*_(10)_ = 15.02, *p* < 0.0001, unpaired *t* test) show increased levels of oxidative damage around amyloid plaques in AD compared with NDAN individuals. Original magnification, 60×. Scale bar, 30 µm. Values are expressed as the mean ± SD. ****p < 0.0001. ***B***, ***B′***, Immunostaining of 8-oxo-dG and Aβ showing significant high levels of oxidative damage in AD subjects compared with control and NDAN subjects even far from Aβ plaques. Statistical analyses were made using one-way ANOVA (IntDens: *F*_(2,15)_ = 122.1, *p* < 0.000; count: *F*_(2,15)_ = 42.34, *p* < 0.0001) following Tukey's multiple-comparisons test. Original magnification 60×. Scale bar, 30 µm. Values are expressed as the mean ± SD. ****p < 0.0001. ns, not significant.

To determine whether the levels of oxidative damage were associated with amyloid pathology, we analyzed the levels of 8-oxo-dG in relation to the accumulation of neurotoxic Aβ peptide (Fig. 3). A double staining of 8-oxo-dG in combination with an anti-Aβ antibody was performed either around or far from Aβ plaques (Fig. 3*A*,*B*). The quantitative analyses of the immunoreactivity (AD vs NDAN, *p* < 0.0001) and the count of 8-oxo-dG^+^ cells (AD vs NDAN, *p* < 0.0001) showed a significant increase of oxidative damage in AD patients compared with NDAN subjects in the proximity of Aβ plaques (Fig. 3*A*,*A′*). Similarly, when areas far from plaques were considered, NDAN subjects showed significantly lower immunoreactivity levels of 8-oxo-dG, most comparable to control individuals (Fig. 3*B*,*B'*) in both of the analyses performed (IntDens: ctrl vs AD, *p* < 0.0001; AD vs NDAN, *p* < 0.0001; ctrl vs NDAN, *p* = 0.7978; count: ctrl vs AD, *p* < 0.0001; AD vs NDAN, *p* < 0.0001; ctrl vs NDAN, *p* = 0.8616).

**Figure 4.**
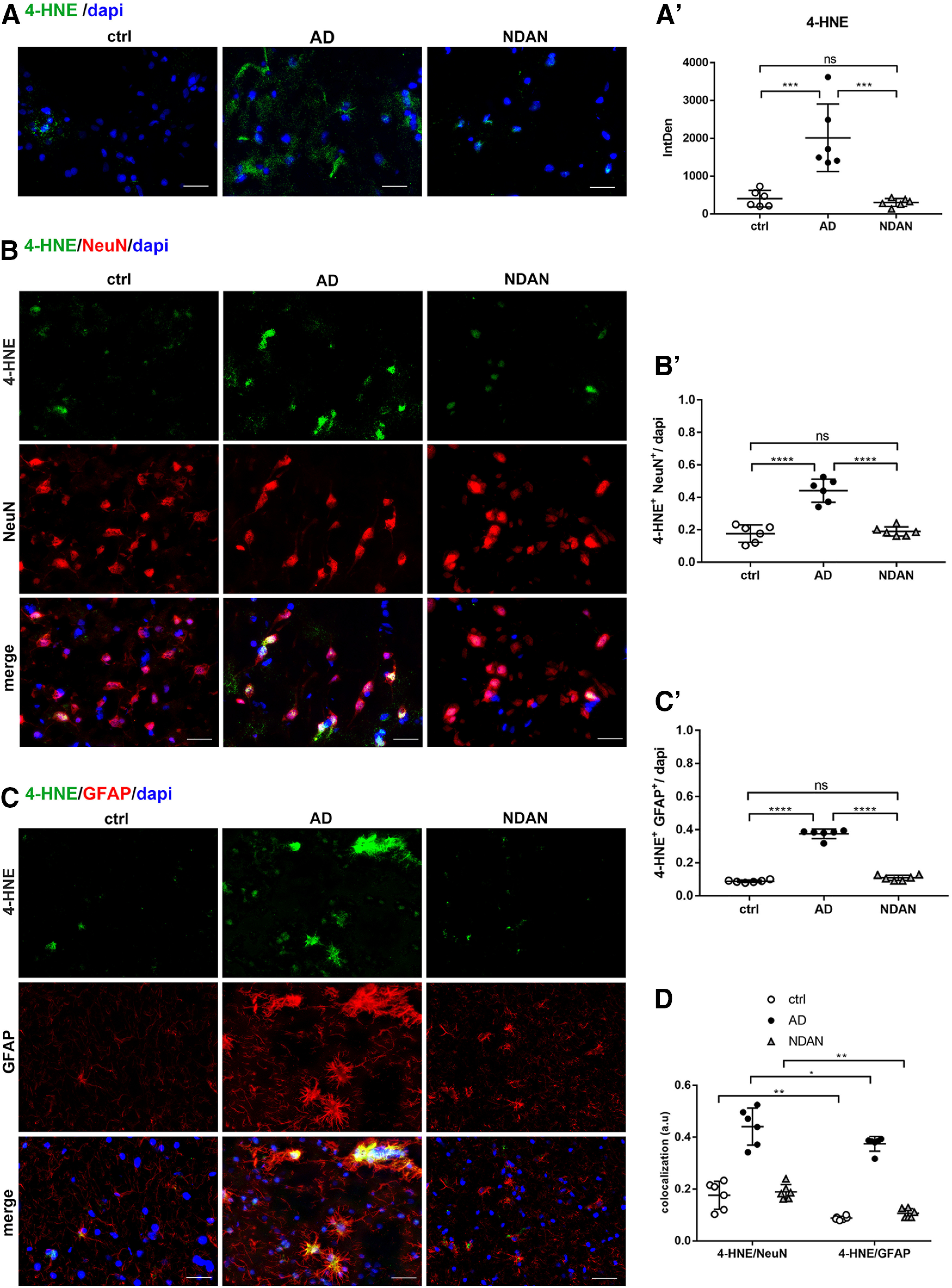
***A***, ***A′***, 4-HNE expression and distribution in frontal cortex of control, AD, and NDAN subjects. Immunolocalization of 4-HNE and quantitative analysis of IF images showing increased levels of the lipid peroxidation marker in AD brains and low levels in NDAN subjects, compared with control subjects. Original magnification, 60×. Scale bar, 30 µm. Statistical analyses were made using one-way ANOVA (*F*_(2,15)_ = 19.44, *p* < 0.0001) following Tukey's test multiple-comparisons test. Values are expressed as the mean ± SD. ***p < 0.001. ***B–D***, 4-HNE expression and distribution in frontal cortex neurons and astrocytes of control, AD, and NDAN subjects. ***B***, ***B′***, Double IF of 4-HNE (green) in combination with NeuN (red) shows high levels of oxidative damage in AD neurons. NDAN neurons demonstrate low levels of lipid peroxidation marker. Magnification, 60×. Scale bar, 30 µm. The quantitative analysis of IF images shows a significantly higher levels of oxidative damage marker in AD neurons. Statistical analyses were made using one-way ANOVA (*F*_(2,15)_ = 45.43, *p* < 0.0001) following Tukey's test multiple-comparisons test. Values are expressed as the mean ± SD. ****p < 0.0001. ***C***, Double IF of 4-HNE (green) in combination with GFAP (red) showing high levels of oxidative damage to astrocytes in AD subjects compared with control and NDAN subjects, although less than in neurons. Magnification, 60×. Scale bar, 30 µm. ***C′***, The quantitative analysis of IF images shows a significantly higher levels of the oxidative damage marker in AD, while NDAN and control astrocytes displayed comparable levels of damage. Statistical analyses were made using one-way ANOVA (*F*_(2,15)_ = 407.9, *p* < 0.0001) following Tukey's test multiple-comparisons test. Values are expressed as the mean ± SD. ****p < 0.0001. ***D***, The analysis demonstrates the significant slightly higher distribution of lipid peroxidation end product in AD neurons versus astrocytes, and the relatively higher resistance of astrocytes to oxidative damage in NDAN. Statistical analyses were made using two-way ANOVA (*F*_(2,30)_ = 172.5, *p* < 0.0001). Values are expressed as the mean ± SD. *p < 0.05; ** *p* < 0.01. ns, not significant.

As a further approach to evaluate oxidative damage, we analyzed the levels and localization of 4-hydroxy-2-nonenal (4-HNE), as a lipid peroxidation end product. 4-HNE is one of the most abundant and cytotoxic lipid-derived alkenals, able to readily react with various cellular components, such as DNA, proteins, and other molecules (Di Domenico et al., 2017). The immunofluorescent staining revealed both cytoplasmic and nuclear localization, possibly indicating the formation of 4-HNE adducts with DNA and/or proteins with significantly higher levels in AD than in control subjects and NDAN individuals (ctrl vs AD, *p* = 0.0003; AD vs NDAN, *p* = 0.0002; Fig. 4*A*,*A′*). Control and NDAN frontal cortices consistently displayed comparable 4-HNE immunoreactivity levels (*p* = 0.9426; Fig. 4*A*,*A′*), similar to what was observed for 8-oxo-dG staining. To investigate either the neuronal or astroglial localization of 4-HNE, we performed double IF with NeuN and GFAP, respectively. The quantitative analyses indicated that in AD frontal cortices, both neurons (Fig. 4*B*,*B*′; ctrl vs AD, *p* < 0.0001; AD vs NDAN, *p* < 0.0001) and astrocytes (Fig. 4*C*,*C′*; ctrl vs AD, *p* < 0.0001; AD vs NDAN, *p* < 0.0001) displayed higher 4-HNE immunoreactivity levels with respect to control subjects and NDAN individuals. Also, in this case, GFAP immunoreactivity appeared especially strong in AD samples, confirming the ongoing astrogliosis. By contrast, in NDAN frontal cortices, no astrogliosis was observed and comparable levels of 4-HNE with controls were detected in both astrocytes (ctrl vs NDAN, *p* = 0.1862) and neurons (ctrl vs NDAN, *p* = 0.9065). Figure 4*D* summarizes the scenario in neurons and astrocytes in the three conditions, highlighting that the production of 4-HNE following lipid peroxidation and the possible formation of adducts, are highly distributed in AD neurons and astrocytes. Interestingly, in NDAN subjects the oxidative damage, even if at much lower levels of AD, predominantly occurs in neurons, while astrocytes appear to be more resistant, showing a significantly weaker staining for 4-HNE (ctrl vs ctrl, *p* = 0.0041; AD vs AD, *p* = 0.0300; NDAN vs NDAN, *p* = 0.0017).

#### SOD2 distribution in neurons and astrocytes

**Figure 5.**
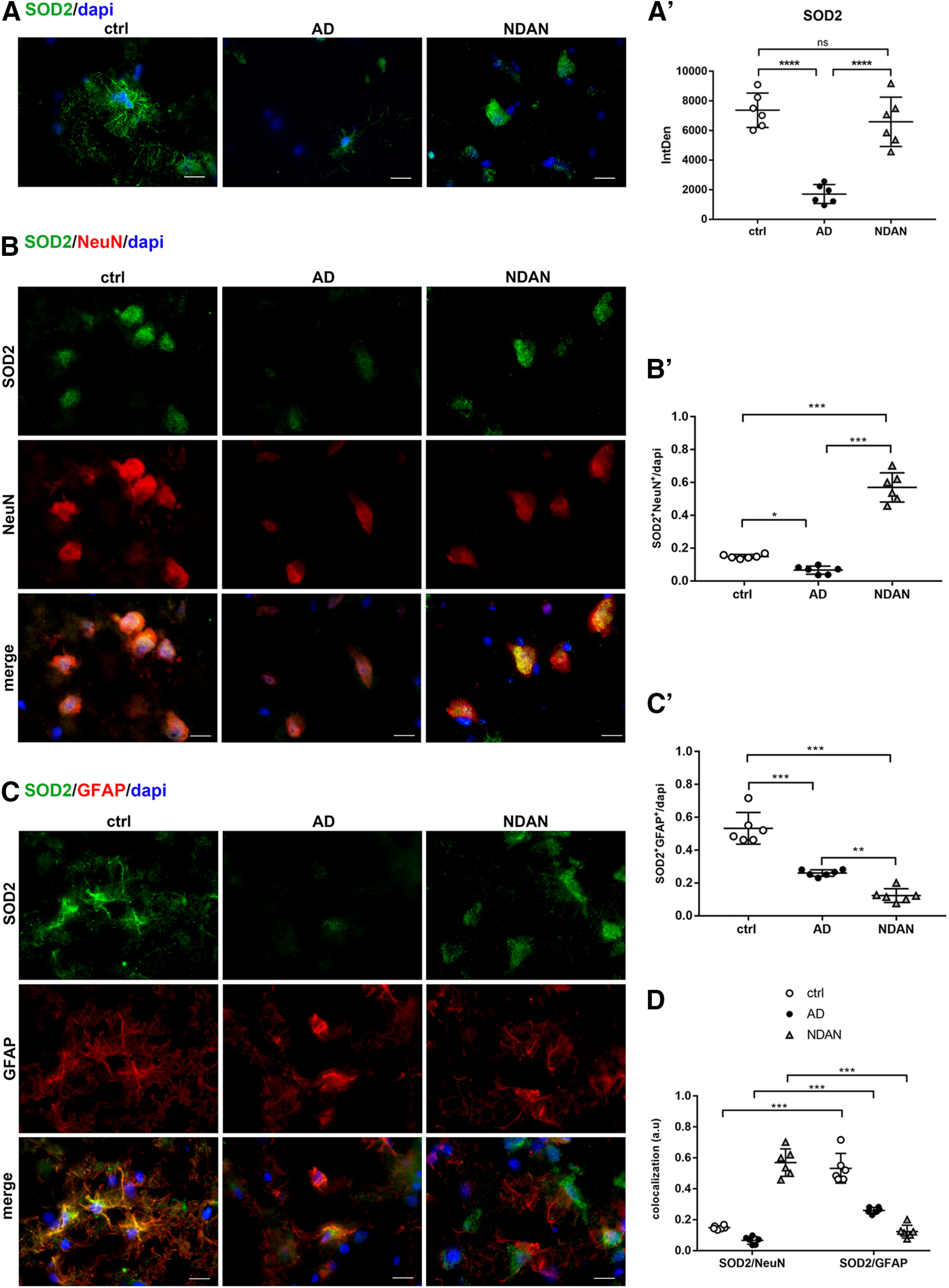
***A***, ***A′***, SOD2 expression in frontal cortex of control, AD, and NDAN subjects. IF images and quantitative analyses showing a significant downregulation of SOD2 in AD patients and preserved levels of SOD2 in NDAN individuals, compared with control subjects. Magnification, 60×. Scale bar, 30 µm. Statistical analyses were made using one-way ANOVA (*F*_(2,15)_ = 30.82, *p* < 0.0001) following Tukey's test multiple-comparisons test. Values are expressed as the mean ± SD. ****p < 0.0001. ***B–D***, SOD2 expression and distribution in frontal cortex neurons and astrocytes of control, AD, and NDAN subjects. ***B***, ***B′***, Double IF of SOD2 (green) in combination with NeuN (red) and quantitative analysis showing significant low levels of the antioxidant enzyme in AD neurons and significantly higher levels in NDAN neurons, compared with control. Magnification 60×. Scale bar, 30 µm. Statistical analyses were made using one-way ANOVA (*F*_(2,15)_ = 151.8, *p* < 0.0001) following Tukey's test multiple-comparisons test. Values are expressed as the mean ± SD. *p < 0.05; ****p < 0.0001. ***C***, ***C′***, Double IF of SOD2 (green) in combination with GFAP (red), and quantitative analysis of images showing the downregulation of the antioxidant enzyme in AD and NDAN, while in AD brains SOD2 mainly localizes to astrocytes. Magnification, 60×. Scale bar, 30 µm. Statistical analyses were made using one-way ANOVA (*F*_(2,15)_ = 68.34, *p* < 0.0001) following Tukey's test multiple-comparisons test. Values are expressed as the mean ± SD. **p < 0.01; ****p < 0.0001. ***D***, The diagram shows an impairment of the antioxidant response in AD subjects and a preserved scavenging system in NDAN. Significantly higher levels of SOD2 in neurons and astrocytes of NDAN and AD, respectively, are highlighted. Statistical analyses were made using two-way ANOVA (*F*_(2,30)_ = 39, *p* < 0.0001). Values are expressed as the mean ± SD. ****p < 0.0001. ns, not significant.

The study of oxidative damage levels prompted us to investigate the antioxidant response status. Particularly, given the well established role of mitochondrial dysfunction as one of the central cytopathologies of AD (Reddy, 2014; Sweeney and Song, 2016; Swerdlow, 2018; Perez Ortiz and Swerdlow, 2019), we analyzed the expression of the mitochondrial O_2_^–.^-scavenging enzyme SOD2 in frontal cortices from AD, NDAN, and normally aged individuals. Quantitative analysis of IF images revealed that SOD2 was significantly downregulated in AD frontal cortex, compared with control patients (ctrl vs AD, *p* < 0.0001). On the other hand, NDAN subjects showed overall normal levels of SOD2 (ctrl vs NDAN, *p* = 0.4712; AD vs NDAN, *p* < 0.0001; Fig. 5*A*,*A′*). In view of the synapses as regions rich in mitochondria, we evaluated the expression of SOD2 in synaptosomes isolated from frontal cortices of control subjects, AD individuals, and NDAN individuals. The analyses conducted either on protein extracts from single individual synaptosomal fraction or on pooled extracts confirmed the morphologic observations showing significantly lower levels of SOD2 in AD individuals compared control and NDAN individuals (Extended Data Fig. 5-1).

10.1523/JNEUROSCI.0295-20.2020.f5-1 Figure 5-1 SOD2 protein levels in synaptosomes. Wb analyses performed on synaptosomal fractions showing significantly decreased levels of SOD2 in AD patients compared with NDAN and control subjects. ***A***, ***B***, The analyses conducted either on protein extracts from single individual synaptosomal fraction (***A***; ctrl vs AD, *p* = 0.0367; ctrl vs NDAN, *p* = 0.9987; AD vs NDAN, *p* = 0.0404) or on pooled extracts (***B***; ctrl vs AD, *p* = 0.0011; ctrl vs NDAN, *p* = 0.9988; AD vs NDAN, *p* = 0.0012) confirmed the preserved antioxidant content in NDAN individuals. Statistical analyses were made using one-way ANOVA (***A***: *F*_(2,18)_ = 4.799, *p* = 0.0214; ***B***: *F*_(2,9)_ = 19.21, *p* = 0.0006) following Tukey's multiple-comparisons test. Values are expressed as fold change as a function of SYN ± SD (*n* = 7/group; 3 technical replicates). *p < 0.05; **p < 0.01. Download Figure 5-1, TIF file.

To properly investigate its neuronal and astroglial distribution, we performed double-IF staining for SOD2 in combination with either NeuN or GFAP (Fig. 5*B*,*C*). Intriguingly, while in control and AD brains the enzyme mainly localized to glial cells (Fig. 5*C*,*C′*; ctrl vs AD, *p* < 0.0001; ctrl vs NDAN, *p* < 0.0001; AD vs NDAN, *p* = 0.0043), in NDAN samples, predominantly neuronal localization was detected (Fig. 5*B*,*B′*; ctrl vs AD, *p* = 0.0428; ctrl vs NDAN, *p* < 0.0001; AD vs NDAN, *p* < 0.0001). Figure 5*D* summarizes the expression and distribution of SOD2 in neurons and astrocytes in all analyzed conditions. While in control and AD brains, astrocytes appear to be more protected than neurons against oxidative challenge, the reverse is true for NDAN brains, where neurons are specifically endowed with high levels of SOD2, even higher than those detected in astrocytes. Figure 5*D* highlights the higher expression of SOD2 in NDAN neurons, suggesting that these individuals could be endowed with a preserved antioxidant response able to counteract redox imbalance (ctrl vs ctrl, *p* < 0.0001; AD vs AD, *p* < 0.0001; NDAN vs NDAN, *p* < 0.0001).

#### Redox sensors: PGC1α and PPARα distribution in neurons and astrocytes

**Figure 6.**
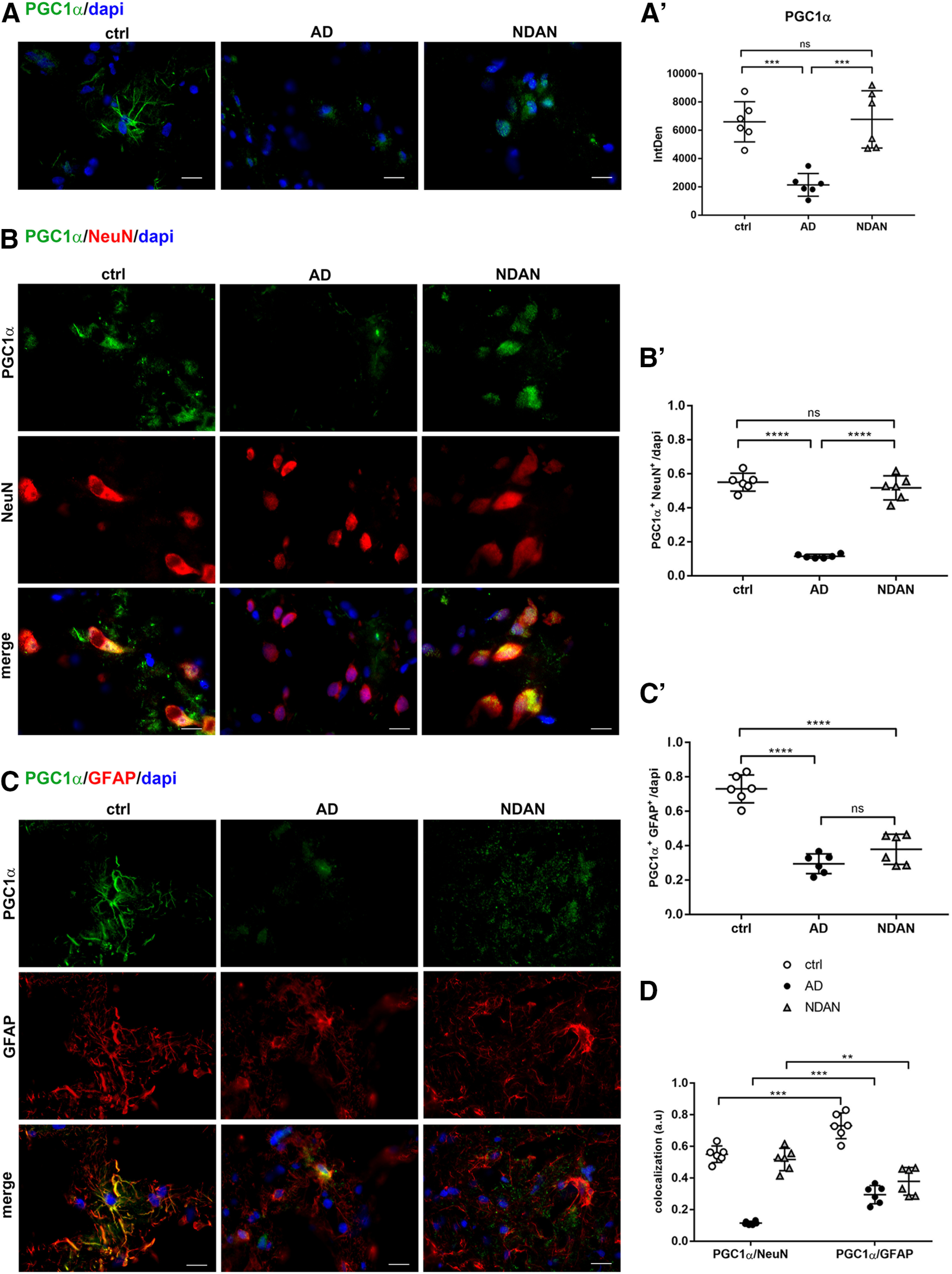
***A***, ***A′***, PGC1α expression in frontal cortex of control, AD, and NDAN subjects. The quantitative analyses of the IF images showing a downregulation of PGC1α in AD and preserved levels in NDAN subjects. Magnification, 60×. Scale bar, 30 µm. Statistical analyses were made using one-way ANOVA (*F*_(2,15)_ = 18.4, *p* < 0.0001) following Tukey's test multiple-comparisons test. Values are expressed as the mean ± SD. ***p < 0.001. ***B–D***, PGC1α expression and distribution in frontal cortex neurons and astrocytes of control, AD, and NDAN subjects. ***B***, ***B′***, Double IF of PGC1α (green) in combination with NeuN (red) showing significantly lower levels of the transcription factor in AD neurons and preserved levels in NDAN neurons. Magnification, 60×. Scale bar, 30 µm. Quantitative analysis of IF images shows significantly higher levels of PGC1α in NDAN neurons compared with AD neurons. Statistical analyses were made using one-way ANOVA (*F*_(2,15)_ = 134, *p* < 0.0001) following Tukey's test multiple-comparisons test. Values are expressed as the mean ± SD. ****p < 0.0001. ***C***, ***C′***, Double IF of PGC1α (green) in combination with GFAP (red) and quantitative analysis showing the downregulation of the transcription factor in AD and NDAN astrocytes compared with controls. Magnification, 60×. Scale bar, 30 µm. Statistical analyses were made using one-way ANOVA (*F*_(2,15)_ = 54.69, *p* < 0.0001) following Tukey's multiple-comparisons test. Values are expressed as the mean ± SD. ****p < 0.0001. ***D***, The analysis shows a downregulation of PGC1α in AD frontal cortex although with a prevalent localization in astrocytes compared with neurons. Conversely, NDAN and control astrocytes display comparable levels of PGC1α, and a significant increase in neurons. Statistical analyses were made using two-way ANOVA (*F*_(2,30)_ = 134.8, *p* < 0.0001). Values are expressed as the mean ± SD. **p < 0.01; ***p < 0.001. ns, not significant.

We analyzed the expression of PGC1α as a key regulator of the antioxidant response involved in the transcriptional activity of several genes (i.e., SOD2; St-Pierre et al., 2006; Aquilano et al., 2013). Quantitative analysis of immunofluorescence microscopy images showed lower expression of PGC1α in AD individuals, while NDAN individuals displayed levels similar to those of control individuals (ctrl vs AD, *p* = 0.0003; ctrl vs NDAN, *p* = 0.9785; AD vs NDAN, 0.0002; Fig. 6*A*,*A′*).

The staining was mostly present in the astroglial population in both control subjects and AD patients (Fig. 6*C*,*C′*; ctrl vs AD, *p* < 0.0001; ctrl vs NDAN, *p* < 0.0001; AD vs NDAN, *p* = 0.1687). Conversely, in NDAN subjects, PGC1α was mainly localized in neurons (Fig. 6*B*,*B′*; ctrl vs AD, *p* < 0.0001; ctrl vs NDAN, *p* = 0.5076; AD vs NDAN, *p* < 0.0001). Figure 6*D* summarizes the expression and distribution of PGC1α in neurons and astrocytes in all analyzed conditions (ctrl vs ctrl, *p* = 0.0001; AD vs AD, *p* = 0.0001; NDAN vs NDAN, *p* = 0.0028). Wb analyses conducted on total protein extracts confirmed the significant downregulation of PGC1α in AD frontal cortex compared with control subjects and NDAN individuals, with the latter displaying levels similar to those of control subjects (Extended Data Fig. 6-1).

10.1523/JNEUROSCI.0295-20.2020.f6-1 Figure 6-1 PGC1α expression in cortical total protein extracts. Wb analyses performed on total protein extracts showing a significant decrease of PGC1α in AD cortical samples compared with those from control and NDAN samples. ***A***, ***B***, The analyses conducted either on total protein extracts from single individuals (***A***: ctrl vs AD, *p* = 0.2094; ctrl vs NDAN, *p* = 0.3962; AD vs NDAN, *p* = 0.0163) or on pooled extracts (***B***: ctrl vs AD, *p* = 0.0426; ctrl vs NDAN, *p* = 0.7228; AD vs NDAN, *p* = 0.0169) indicate higher levels of PGC1α in NDAN subjects compared with AD patients. Statistical analyses were made using one-way ANOVA (***A***: *F*_(2,18)_ = 4.829, *p* = 0.0209; ***B***: *F*_(2,6)_ = 8.924, *p* = 0.0159) following Tukey's multiple-comparisons test. Values are expressed as the fold change as function of ACTB ± SD (*n* = 7/group; 3 technical replicates). *p < 0.05. Download Figure 6-1, TIF file.

**Figure 7.**
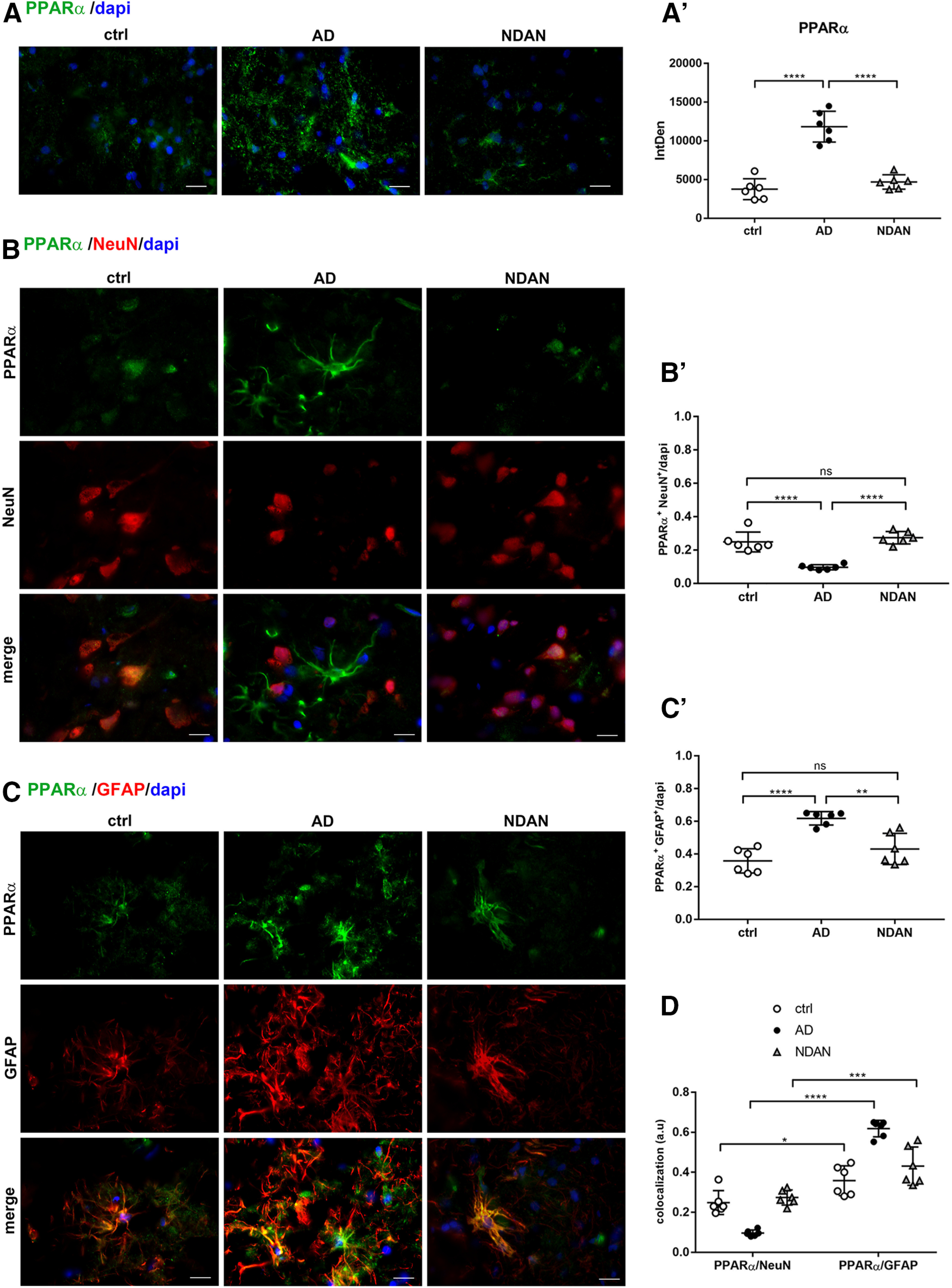
***A***, ***A′***, PPARα expression in frontal cortex of control, AD, and NDAN subjects. The quantitative analyses of the IF images showing upregulation of PPARα in AD compared with control subjects. NDAN and control subjects show comparable levels of the nuclear receptor. Magnification, 60×. Scale bar, 30 µm. Statistical analyses were made using one-way ANOVA (*F*_(2,15)_ = 52.78, *p* < 0.0001) following Tukey's test multiple-comparisons test. Values are expressed as the mean ± SD. ****p < 0.0001. ***B–D***, PPARα expression and distribution in frontal cortex neurons and astrocytes of control, AD, and NDAN subjects. ***B***, ***B′***, Double IF of PPARα (green) in combination with NeuN (red) showing significant downregulation of the nuclear receptor in AD neurons. Magnification, 60×. Scale bar, 30 µm. Quantitative analysis of IF images showing a similar neuronal localization of PPARα in NDAN compared with AD subjects. Statistical analyses were made using one-way ANOVA (*F*_(2,15)_ = 31.94, *p* < 0.0001) following Tukey's test multiple-comparisons test. Values are expressed as the mean ± SD. ****p < 0.0001. ***C***, ***C′***, Double IF of PPARα (green) in combination with GFAP (red) and quantitative analysis showing a predominant localization of the nuclear receptor in AD astrocytes, while NDAN and control astrocytes display comparable levels of PPARα. Magnification, 60×. Scale bar, 30 µm. Statistical analyses were made using one-way ANOVA (*F*_(2,15)_ = 19.85, *p* < 0.0001) following Tukey's test multiple-comparisons test. Values are expressed as the mean ± SD. **p < 0.01; ****p < 0.0001. ***D***, The analysis shows the significant upregulation of PPARα in AD astrocytes. NDAN and control subjects display comparable levels of PPARα in both neurons and astrocytes. Statistical analyses were made using two-way ANOVA (*F*_(2,30)_ = 42.54, *p* < 0.0001). Values are expressed as the mean ± SD. *p < 0.05; ***p < 0.001; ****p < 0.0001. ns, not significant.

Based on the role of PGC1α as a coactivator of PPARα, we further analyzed the expression and distribution of the latter nuclear receptor, as an important oxidative stress sensor and a regulator of energy metabolism. Extensive analysis of PPARα-immunoreacted sections revealed a significantly higher positivity in AD frontal cortex compared with control subjects (ctrl vs AD, *p* < 0.0001). Conversely, though similar to what was observed for PGC1α, NDAN brains displayed levels of PPARα comparable to those of control brains (ctrl vs NDAN, *p* = 0.5325; AD vs NDAN, *p* < 0.0001; Fig. 7*A*,*A′*). Wb analyses conducted on control, AD, and NDAN total lysates confirmed the morphologic observations, showing a significant increase of PPARα expression in AD individuals compared with control subjects and NDAN individuals (Extended Data Fig. 7-1).

10.1523/JNEUROSCI.0295-20.2020.f7-1 Figure 7-1 PPARα expression in cortical total protein extracts. Wb analyses performed on total protein extracts showing significant increase of PPARα in AD cortical samples compared with control and NDAN subjects. ***A***, ***B***, The analyses conducted either on total protein extracts from single individuals (***A***: ctrl vs AD, *p* = 0.0121; ctrl vs NDAN, *p* = 0.1106; AD vs NDAN, *p* = 0.5266) or on pooled total protein extracts (***B***: ctrl vs AD, *p* = 0.0223 ctrl vs NDAN, *p* = 0.9999; AD vs NDAN, *p* = 0.0227) indicate higher levels of PPARα in AD patients. Statistical analyses were made using one-way ANOVA (***A***: *F*_(2,18)_ = 5.412, *p* = 0.0144; ***B***: *F*_(2,6)_ = 9.317, *p* = 0.0144) following Tukey's multiple-comparisons test. Values are expressed as the fold change as a function of ACTB ± SD (*n* = 7/group; 3 technical replicates). *p < 0.05. Download Figure 7-1, TIF file.

Somewhat surprisingly, the localization of the nuclear receptor PPARα appeared as both nuclear and cytosolic, regardless of the patient group (control subjects, and AD and NDAN individuals; Fig. 7*A*). Double immunofluorescence demonstrated prevalent colocalization of PPARα with the astroglial marker GFAP (Fig. 7*C*,*C*′), compared with the neuron-specific marker NeuN (Fig. 7*B*,*B′*). While this general trend was shared by all groups, a significant increase in AD astrocytes (ctrl vs AD, *p* < 0.0001) accompanied by a decrease in neurons was observed (ctrl vs AD, *p* < 0.0001). Compared with AD patients, NDAN patients interestingly showed fainter glial immunoreactivity (ctrl vs NDAN, *p* = 0.2359; AD vs NDAN, *p* = 0.0014; Fig. 7*C*,*C′*) and higher neuronal expression (ctrl vs NDAN, *p* = 0.5549; AD vs NDAN, *p* < 0.0001; Fig. 7*B*,*B′*). Figure 7*D* displays the relative values of the colocalization of PPARα in neurons and astrocytes in all analyzed conditions (ctrl vs ctrl, *p* = 0.0107; AD vs AD, *p* < 0.0001; NDAN vs NDAN, *p* = 0.0003).

#### CAT distribution in neurons and astrocytes

**Figure 8.**
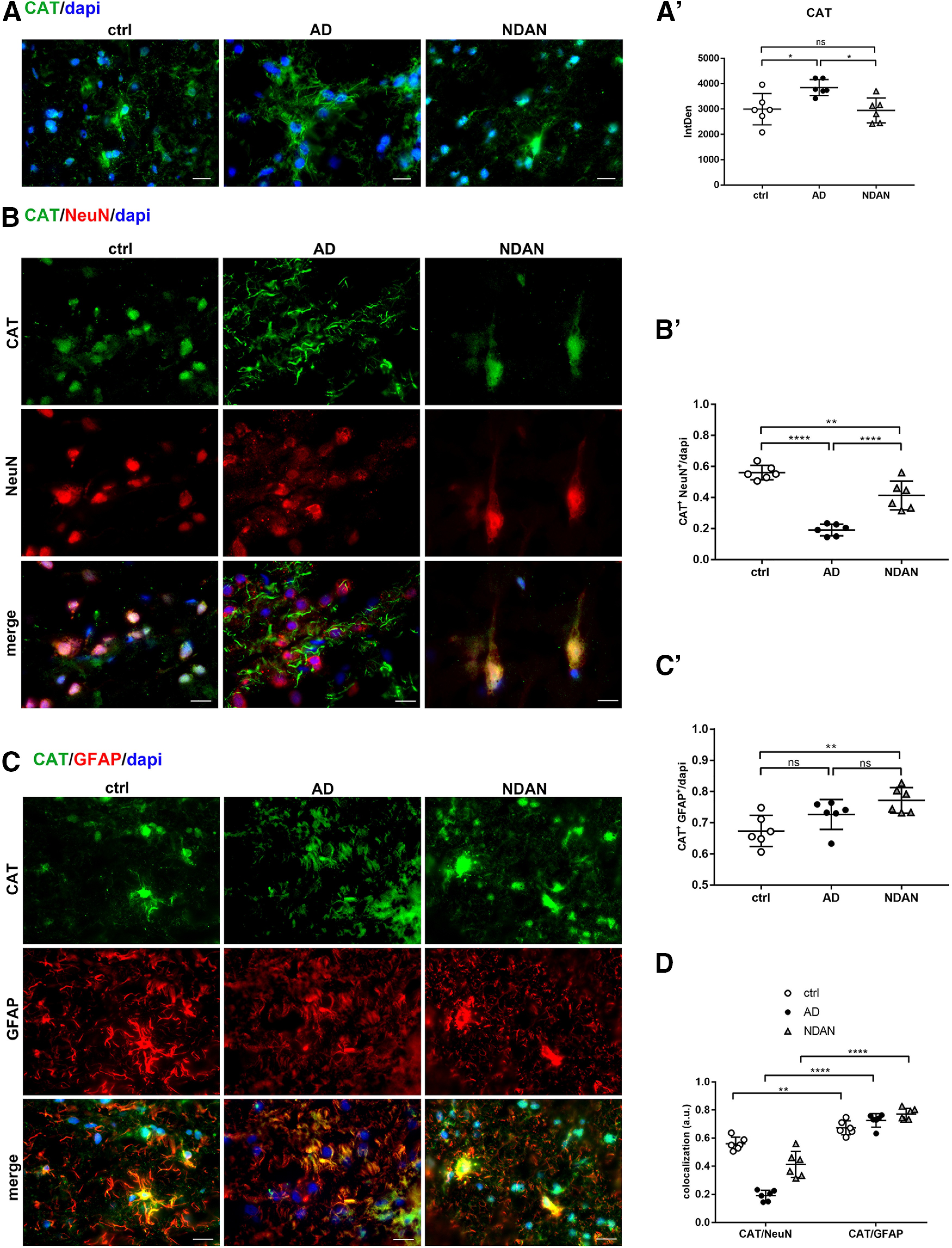
***A***, ***A′***, CAT expression in frontal cortex of control, AD, and NDAN subjects. ***A***, The quantitative analyses of the IF images showing upregulation of CAT in AD compared with control subjects. NDAN and control subjects show comparable levels of the antioxidant enzyme. Magnification, 60×. Scale bar, 30 µm. Statistical analyses were made using one-way ANOVA (*F*_(2,15)_ = 6.387, *p* = 0.0099) following Tukey's test multiple-comparisons test. Values are expressed as the mean ± SD. *p < 0.05. ***B–D***, CAT expression and distribution in frontal cortex neurons and astrocytes of control, AD, and NDAN subjects. ***B***, ***B′***, Double IF of CAT (green) in combination with NeuN (red) and quantitative analysis showing significant lower levels of the antioxidant enzyme in AD neurons compared with control neurons, and significantly higher levels in NDAN neurons compared with AD neurons. Magnification, 60×. Scale bar, 30 µm. Statistical analyses were made using one-way ANOVA (*F*_(2,15)_ = 50.94, *p* < 0.0001) following Tukey's test multiple-comparisons test. Values are expressed as the mean ± SD. **p < 0.01; ****p < 0.0001. ***C***, ***C′***, Double IF of CAT (green) in combination with GFAP (red) showing predominant nuclear localization of the H_2_O_2_-scavenging enzymes in NDAN patients. Quantitative analysis of images showing no significant changes of CAT expression in astrocytes. Magnification, 60×. Scale bar, 30 µm. Statistical analyses were made using one-way ANOVA (*F*_(2,15)_ = 6.752, **p = 0.0081) following Tukey's multiple-comparisons test. Values are expressed as the mean ± SD. **p < 0.01. ***D***, The diagram shows a significantly predominant localization of CAT in astrocytes rather than in neurons in all the three considered conditions. Statistical analyses were made using two-way ANOVA (*F*_(2,30)_ = 28, *p* < 0.0001). Values are expressed as the mean ± SD. **p < 0.01; ****p < 0.0001. ns, not significant.

The increased expression of PPARα in AD patients prompted us to analyze the distribution of one of the major peroxisomal proteins, the scavenging enzyme CAT, whose transcription is driven by both PPARα and PGC1α. We observed significantly higher expression of CAT in AD patients, while no significant differences were detected between control and NDAN subjects (ctrl vs AD, *p* = 0.0229; ctrl vs NDAN, *p* = 0.9823; AD vs NDAN, *p* = 0.0161; Fig. 8*A*,*A′*). Wb analyses performed on cytosolic fractions showed the same trend of IF experiments, confirming a significant increase of CAT in AD patients and no significant variations between control subjects and NDAN individuals (Extended Data Fig. 8-1). Interestingly, the highest levels of the peroxisomal enzyme in both AD and NDAN patients were found in astrocytes (ctrl vs AD, *p* = 0.1536; ctrl vs NDAN, *p* = 0.0060; AD vs NDAN, *p* = 0.2376; Fig. 8*C*,*C′*), whereas a significant downregulation of neuronal CAT was detected in AD individuals (ctrl vs AD, *p* < 0.0001; ctrl vs NDAN, 0.0032 AD vs NDAN, *p* < 0.0001; Fig. 8*B*,*B′*). Figure 8*D* summarizes the expression and distribution of CAT in neurons and astrocytes in all of the analyzed conditions (ctrl vs ctrl, *p* = 0.0044; AD vs AD, *p* < 0.0001; NDAN vs NDAN, *p* < 0.0001). Somewhat surprisingly, a prominent nuclear rather than the canonical cytosolic localization of the scavenger enzyme was detected in NDAN patients, as shown in Figure 8, *B* and *C*.

10.1523/JNEUROSCI.0295-20.2020.f8-1 Figure 8-1 CAT expression levels in cytosolic fraction. Wb analyses performed on cytosolic fraction showing a significant increase of CAT in AD frontal cortices compared with those of control and NDAN subjects. ***A***, ***B***, Analyses conducted either on cytosolic protein extracts from single individuals (***A***: ctrl vs AD, *p* = 0.0297; ctrl vs NDAN, *p* = 0.5300; AD vs NDAN, *p* = 0.2265) or on pooled cytosolic protein extracts (***B***: ctrl vs AD, *p* = 0.0089; ctrl vs NDAN, *p* = 0.1711; AD vs NDAN, *p* = 0.1867) indicate higher levels of CAT in AD patients. Statistical analyses were made using one-way ANOVA (***A***: *F*_(2,18)_ = 4.012, *p* = 0.0362; ***B***: *F*_(2,9)_ = 7.659, *p* = 0.0114) following Tukey's multiple-comparisons test. Values are expressed as the fold change as a function of ACTB ± SD (*n* = 7/group; 4 technical replicates). *p < 0.05; **p < 0.01. Download Figure 8-1, TIF file.

### Regulation of PGC1α via miRNA-485

**Figure 9.**
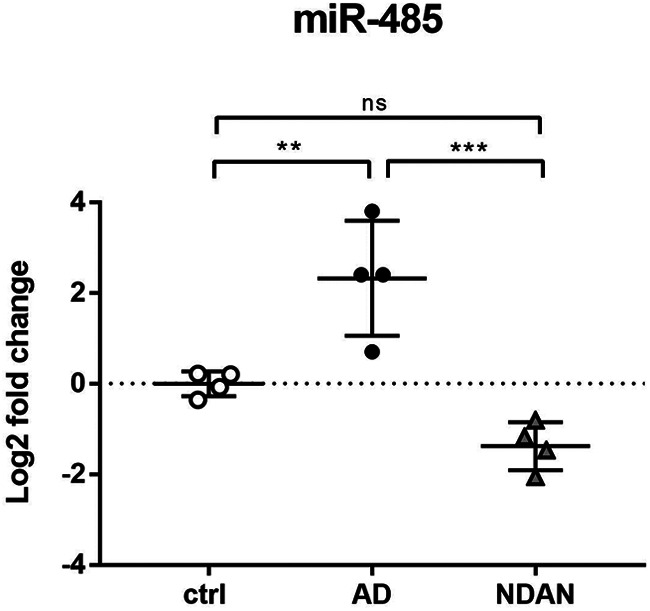
Regulation of PGC1α via miRNA-485. Assessment of miRNA-485 levels in frontal cortices of control, AD, and NDAN subjects by real-time PCR shows an increase in AD, whereas a significant decrease in NDAN versus control is observed. Statistical analyses were made using one-way ANOVA (*F*_(2,9)_ = 21.46, *p* = 0.0004) following Tukey's multiple-comparisons test. Values are expressed as the mean ± SD. **p < 0.01; ***p < 0.001. ns, not significant.

Given the importance of PGC1α as a key modulator of antioxidant responses and its levels differentially downregulated in AD individuals and preserved in NDAN individuals, we wanted to further investigate its upstream regulators in the frontal cortices of control, AD, and NDAN subjects. To that end, we measured the tissue levels of miRNA-485, which has been shown, although in non-neuronal tissue, to negatively modulate the transcription and expression of PGC1α (Lou et al., 2016). As shown in Figure 9, using quantitative RT-PCR, we found that the expression of miRNA-485 was significantly increased in the cortices of AD individuals compared with control subjects (ctrl vs AD, *p* = 0.007) and NDAN subjects (AD vs NDAN, *p* = 0.003). On the other hand, no significant differences were detected between NDAN individuals and control subjects (ctrl vs NDAN, *p* = 0.0909).

## Discussion

The aim of this work was to investigate the relationship among amyloid overload, oxidative stress, and cellular response elicited by this status. To this purpose, AD, NDAN, and normally aged individuals were comparatively analyzed, focusing on the frontal cortex as one brain region most vulnerable to dementia. This study, highlighting AD-related alterations to pathways regulating cellular redox homeostasis, also sheds light onto the mechanisms allowing NDAN subjects to preserve cognitive functions, despite Aβ toxic insult.

Concerning AD patients, redox imbalance was demonstrated by the increased immunofluorescent distribution of the DNA/RNA oxidative damage marker 8-oxo-dG and lipid peroxidation end product 4-HNE. Interestingly, we observed high levels of oxidative damage to nucleic acids in AD patients in the proximity of Aβ plaques, where oligomers are particularly abundant. These findings are consistent with the well established notion that oligomers are the most toxic species in AD (Selkoe, 2008; Sengupta et al., 2016). These results, supporting the current concept that oxidative stress is a major and early causative factor in AD (Mecocci et al., 1994; Hensley et al., 1995; Markesbery, 1997; Smith et al., 2000; Butterfield et al., 2001; Lauderback et al., 2001; Sayre et al., 2008; Sultana and Butterfield, 2013; Zhao and Zhao, 2013; Bonda et al., 2014; Wang et al., 2014; Kim et al., 2015; Luca et al., 2015; Huang et al., 2016; Jiang et al., 2016; Sanabria-Castro et al., 2017; Cheignon et al., 2018; D'Orio et al., 2018), also correlates with our previous data on Tg2576 mice (Fanelli et al., 2013; Porcellotti et al., 2015). The substantial localization of 8-oxo-dG in the neuronal cytoplasm, already observed in the mouse model (Porcellotti et al., 2015) indicates a prevalence of modifications to mitochondrial nucleic acids or cytosolic RNA, consistent with the well established mitochondrial abnormalities as prominent features of AD (Cai and Tammineni, 2017). On the other hand, the cytosolic and nuclear localization of 4-HNE suggests that this lipid peroxidation end product actively reacts with either cytoplasmic or nuclear proteins, thus increasing the oxidative stress status. Interestingly, the relatively scarce 8-oxo-dG and 4-HNE immunoreactivity in astrocytes suggests that this cell type could be consistently protected from oxidative stress in AD, reflecting cell population specificity, in terms of antioxidant defenses. Indeed, the greater resistance of astroglia possibly relates to their higher content in SOD2, consistent with our findings in aged Tg2576 mice (Porcellotti et al., 2015). Noteworthy, and consistent with the dramatic oxidative damage, we found a significant decrease of total SOD2 levels in AD versus control brains, which are especially sharp in neurons, thus supporting the idea that altered expression of this mitochondrial enzyme is crucial in the progression of AD pathology (Cimini et al., 2009; Massaad et al., 2009; Fanelli et al., 2013; Flynn and Melov, 2013; Hroudová et al., 2014; Porcellotti et al., 2015; Majd and Power, 2018; Swerdlow, 2018; Perez Ortiz and Swerdlow, 2019). In relation to these changes, the levels and distributions of the transcription factor PGC1α were investigated. In agreement with the literature (Qin et al., 2009), we found a significant downregulation of PGC1α in AD brains compared with control brains, exactly reflecting the expression and localization of its target gene product SOD2. Indeed, PGC1α was mainly localized in astrocytes, in accordance with the localization observed in aged Tg2576 mice (Porcellotti et al., 2015), and supporting the above hypothesis of cell type-based antioxidant response ability. As PGC1α regulates mitochondrial and peroxisomal biogenesis (Austin and St-Pierre, 2012), a correlation between the oxidative stress observed in AD frontal cortices and dysfunctions of these organelles, likely because of PGC1α downregulation, could be hypothesized (Demarquoy and Le Borgne, 2015; Sweeney and Song, 2016; Wanders et al., 2016). The reason for such decreased expression may well relate to enhanced levels of miRNA-485, as we assessed by quantitative RT-PCR. This molecule has indeed recently been demonstrated to negatively regulate PGC1α (Lou et al., 2016).

We analyzed the expression of PPARα, not only for its synergism with PGC1α, but also in view of its roles in energy metabolism and in the modulation of neuroinflammation in AD (Feige et al., 2006; Fidaleo et al., 2014). Increased levels of this receptor in AD brains, compared with those of control subjects, were detected, suggesting a possible activation of mechanisms to compensate for mitochondrial dysfunction, likely through stimulation of peroxisomal-based energy metabolism. Indeed, in AD patients, the significant increase of CAT, whose transcription is driven by PPARα (Shin et al., 2016), well correlates with this hypothesis. The higher levels of the H_2_O_2_ scavenging enzyme might represent an abortive attempt to cope with the oxidative stress activating peroxisomal detoxifying pathways. The predominant astroglial localization of CAT is consistent with the higher oxidative damage found in AD neurons confirming the hypothesis that astrocytes could be more resistant to oxidative damage being characterized of higher levels of antioxidant enzymes. However, the higher concentration of antioxidant enzymes is unsuccessful due the multiple sources of intracellular and extracellular ROS production (Tönnies and Trushina, 2017; Bodega et al, 2019). Further studies using appropriate markers and biochemical assays are needed to ascertain putative peroxisomal proliferation and/or activation.

Compelling evidence demonstrates that the expression and activity of PPARα are influenced by oxidative stress (Kim and Yang, 2013), and its activation is likely because of oxidized lipids, which may act as specific ligands (Yeldandi et al., 2000). In this context, 4-HNE has been described to act as a PPARα endogenous agonist (Manea et al., 2015). This event, possibly explaining the higher levels of PPARα in AD patients, could, however, represent a double-edged sword. If on the one hand the activation of PPARα could result in peroxisomal proliferation, on the other hand the resulting increased fatty acid β-oxidation might trigger an excessive formation of ROS (Del Río and López-Huertas, 2016; Lismont et al., 2019; Liu et al., 2019). Moreover, the predominantly cytosolic immunostaining and astroglial localization could reflect a nongenomic action of PPARα (Feige et al., 2006), particularly related to its anti-inflammatory role in response to Aβ toxicity. Such interpretation is consistent with the remarkable astrogliosis and consequent neuroinflammation occurring at advanced AD stages (Birch, 2014; Osborn et al., 2016; González-Reyes et al., 2017). It is possible, however, that the augmented expression of PPARα in AD cortex is not sufficient *per se* to exert efficient neuroprotective and anti-inflammatory actions, since the available endogenous ligands may be present at very low concentrations (Roy et al., 2016). Supplementing the brain with appropriate dosage of PPARα agonists may thus be of therapeutic value, especially as a supporting treatment, possibly in combination with antioxidants. Among PPARα ligands, naturally occurring substances (e.g., oleoylethanolamide and palmitoylethanolamide), as well as synthetic molecules (e.g., fibrates), have been proven effective in rescuing neurodegeneration, while promoting neuroregeneration, in a number of *in vitro* and *in vivo* models of neuropathologies (Fidaleo et al., 2014; D'Orio et al., 2018). The feasibility of such trials is encouraged by the current use of several fibrates, in therapeutic protocols against hypercholesterolemia and hyperlipidemia, both of which are recognized as risk factors in AD (Xue-shan et al., 2016; Wong et al., 2017).

A completely different scenario from that of AD patients emerged from the study of NDAN individuals, characterized by lesser susceptibility to oxidative damage, associated with more efficacious antioxidant response. Indeed, analyses performed on NDAN brain samples revealed remarkable similarities with control brains, rather than AD brains. In this context, cognitive reserve could play a key role in contributing to the synaptic resilience observed in NDAN individuals, who may display brain flexibility and adaptability leading to cognitive networks that resist or compensate for the effects of AD- or aging-related changes (Stern et al., 2019).

Interestingly, the very low levels of 8-oxo-dG, found also in the proximity of Aβ plaques, where Aβ oligomers are especially abundant, might be consistent with the idea that NDAN synapses are more resistant to Aβ oligomers (Bjorklund et al., 2012), strengthening the concept of synaptic resilience. Moreover, the relatively low levels of 8-oxo-dG and 4-HNE in NDAN frontal cortex detected in both neurons and astrocytes may well result from preserved antioxidant response mechanisms, including not only SOD2-based scavenging system, but also its major regulator PGC1α. Indeed, unchanged total levels of PGC1α, compared with control subjects, are a hallmark of the NDAN frontal cortex. This peculiarity is likely because of the lack of inhibition by miRNA-485, the expression of which is low in these individuals. It is relevant to point out that PGC1α pattern is mostly because of neuronal contribution, suggesting a specific ability of this cell type in NDAN to activate a compensatory response against superoxide-mediated damage. Accordingly, SOD2 is selectively induced in NDAN neurons, as confirmed also by the high content of SOD2 in NDAN synaptosomes, suggesting the maintenance of mitochondrial homeostasis and integrity, despite Aβ insult. Therefore, energy metabolism is likely preserved in NDAN, and no PPARα activation is required to induce peroxisomal biogenesis, compensating for mitochondrial dysfunction. Consistent with this hypothesis, NDAN individuals and control subjects display comparable PPARα patterns. Consistent with these data, no significant differences were found between control and NDAN individuals in terms of CAT expression supporting the hypothesis that NDAN subjects are characterized by a preserved antioxidant response even against the accumulation of H_2_O_2_ leading to significantly low levels of oxidative stress. The preserved capability to resist the oxidative damage may also correlate with the low levels of 4-HNE, which, at such low levels, have been described as acting as a defense mechanism promoting cell survival and proliferation, as well as antioxidant response *via* an NRF2-mediated pathway (Chen et al., 2005; Breitzig et al., 2016; Di Domenico et al., 2017).

Our results on NDAN individuals thus could demonstrate for the first time that an efficient antioxidant response, possibly involving PGC1α might represent a major mechanism by which these individuals resist the detrimental burden of Aβ, thus preventing cognitive impairment. It should be here mentioned that NDAN display lower levels of amyloid oligomers at postsynaptic sites (Bjorklund et al., 2012). Whether this feature is a cause or a consequence of the preserved antioxidant defense is yet to be addressed. It is possible that a vicious cycle linking Aβ, ROS, oxidative damage, and antioxidant response (Sayre et al., 2008; Sultana and Butterfield, 2013; Zhao and Zhao, 2013; Bonda et al., 2014; Wang et al., 2014; Luca et al., 2015; Huang et al., 2016; Cheignon et al., 2018) is not triggered in NDAN. This aspect, together with the proposed feature of NDAN individuals to display a preserved neurogenesis (Briley et al., 2016), could explain the intactness of cognitive abilities in these subjects, while helping to identify novel targets for an AD cure.

**Figure 10.**
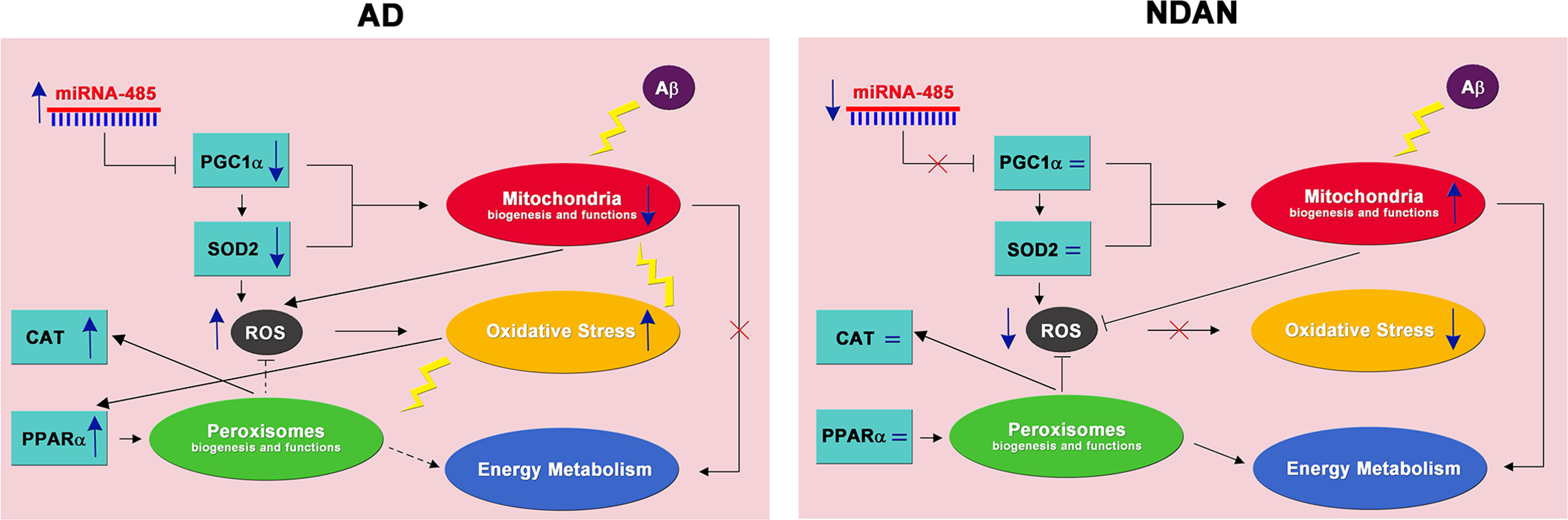
Antioxidant response and oxidative stress in AD and NDAN frontal cortices. Left, Aβ plays a critical role in AD pathogenesis leading to mitochondrial alterations in terms of biogenesis and functions. Downregulation of PGC1α, possibly inhibited by high levels of miRNA-485, and its target gene SOD2 contribute to energy dysmetabolism. Mitochondrial dysfunction and antioxidant response impairment lead to ROS increase and oxidative stress, affecting both mitochondria and peroxisomes (yellow lightning). PPARα increase, in response to redox imbalance, may activate a peroxisomal-based energy metabolism, as well an ROS-detoxifying mechanism (dotted lines), compensating for mitochondrial dysfunction. Right, In the frontal cortex of NDAN subjects, the lack of PGC1α miRNA-485-related inhibition results in unchanged levels of PGC1α and SOD2, and thus preserved antioxidant response and mitochondrial integrity, blunting oxidative damage. This suggests that the activation of a PGC1α-dependent response, to cope with the redox imbalance, is crucial to prevent Aβ-mediated toxicity. The unchanged levels of PPARα keep peroxisomes at a physiological level. Based on this, both mitochondria and peroxisomes cooperate in ROS and energy metabolism.

The present study brings new evidence to confirm the crucial role of redox imbalance in AD pathogenesis, emphasizing the importance of effective antioxidant defenses to cope with Aβ-mediated insult. In this context, the comparative analysis of AD *vs.* NDAN individuals proved especially enlightening in clarifying the role of specific factors. In AD patients, low efficacy of antioxidant response, possibly involving PGC1α as a regulator and SOD2 as an effector, likely allows the vicious cycle linking Aβ, ROS, and oxidative damage to occur, leading to dementia progression. By contrast, the low oxidative damage, correlated with the high content of scavenging systems, observed in the NDAN frontal cortex suggests that the ability to activate a PGC1α-dependent “safety mechanisms” to resist oxidative imbalance might be crucial to prevent Aβ-mediated detrimental effects (Fig. 10). The analyses conducted in a comparative manner in neurons and astrocytes further highlight cell-specific processes to counteract redox imbalance.

These emerging concepts may help to envision neuroprotective therapies aimed at ameliorating defects in antioxidant responses in AD patients. Such treatments might involve either PGC1α induction, possibly by modulation of its inhibitor miRNA-485, or direct PPARα activation, by synthetic or natural ligands. This approach would not only improve ROS metabolism, but, more generally, could induce mitochondrial and/or peroxisomal biogenesis and functions.
