# Supplementary figures and images for: Oxidative Damage and Antioxidant Response in Frontal Cortex of Demented and Nondemented Individuals with Alzheimer's Neuropathology
Source: J Neurosci. 2021 Jan 20;41(3):538–54. doi: 10.1523/JNEUROSCI.0295-20.2020 (PMC7821866; doi:10.1523/JNEUROSCI.0295-20.2020)

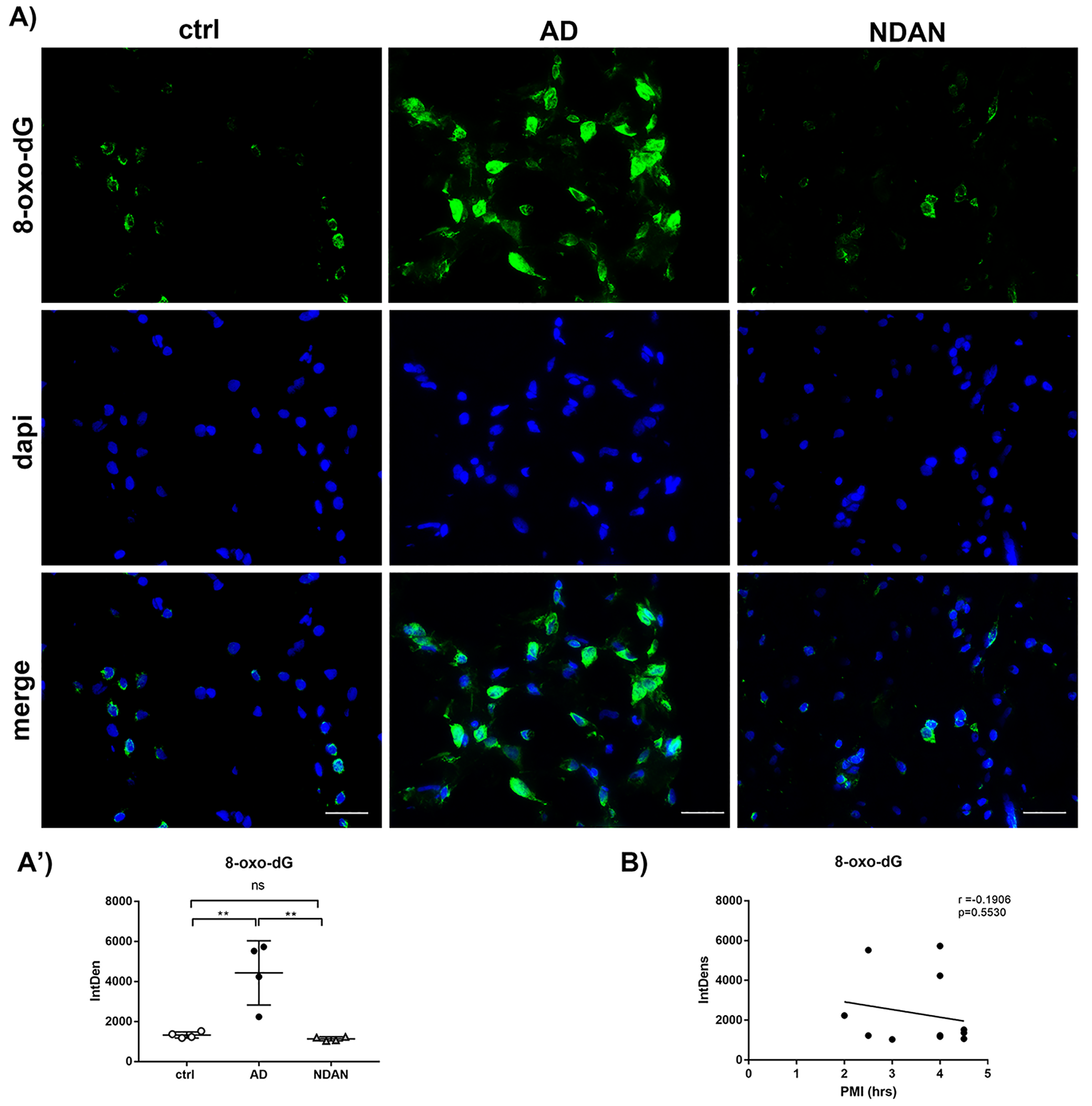

Supplement: Figure 2-1 — 8-Oxo-dG expression and distribution in low-PMI brains. A, A′, 8-Oxo-dG immunostaining and quantitative analyses of control, AD, and NDAN frontal cortices (n = 4) with low PMIs (2–5 h) showing increased oxidative damage in AD subjects compared with control subjects and no significant differences between control and NDAN subjects. Original magnification, 60×. Scale bar, 30 µm. Statistical analyses were made using one-way ANOVA (F(2,9) = 15.71, p = 0.0012), following Tukey's multiple-comparisons test. Values are expressed as the mean ± SD. **p < 0.01. B, A Pearson's correlation test was performed for each measurement against the PMI. Correlation coefficient (r) and p values are noted in the individual plots showing no significant correlation with PMI values. Download Figure 2-1, TIF file. [file ns-JN-RM-0295-20-s01.tif]

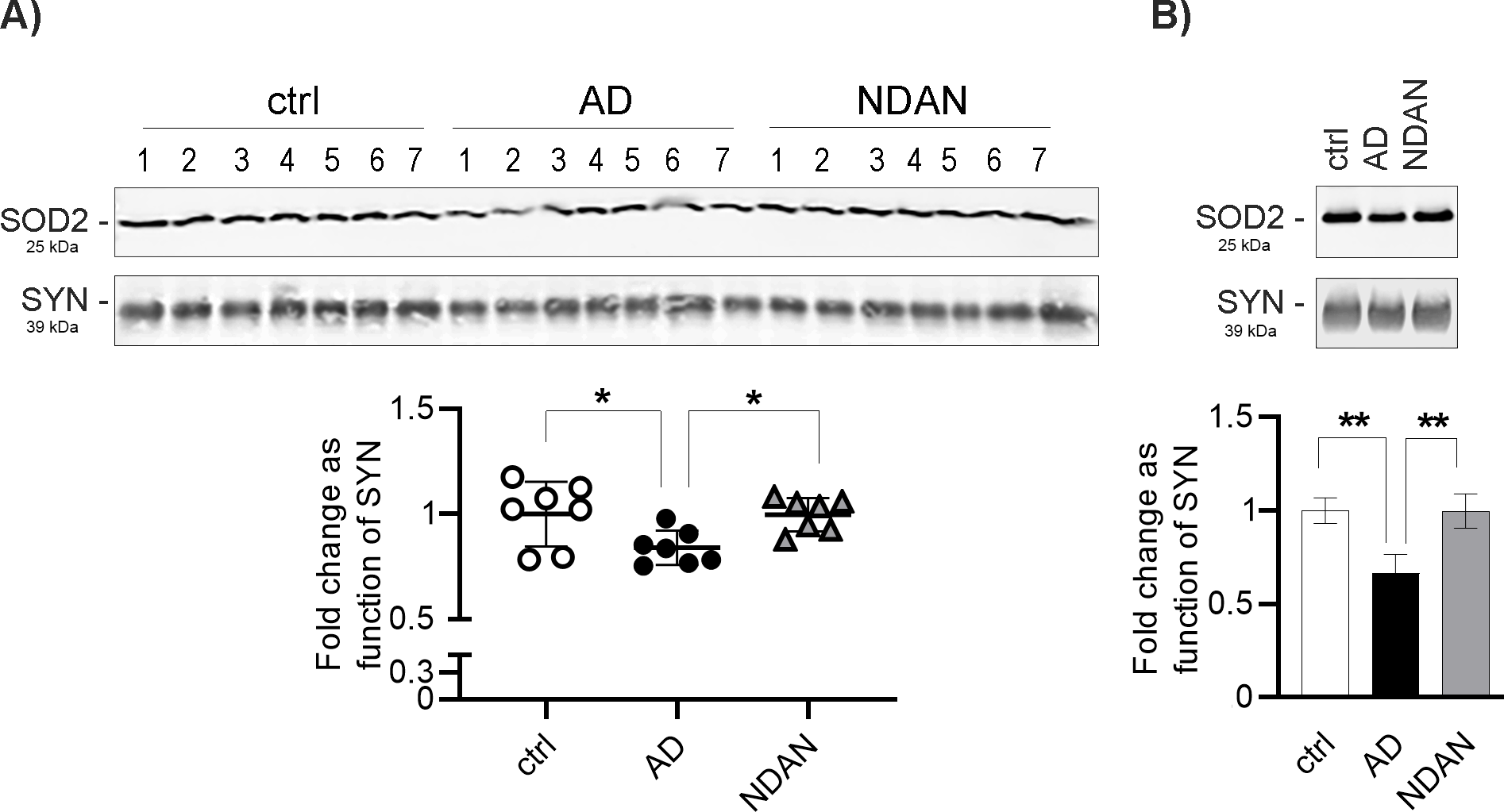

Supplement: Figure 5-1 — SOD2 protein levels in synaptosomes. Wb analyses performed on synaptosomal fractions showing significantly decreased levels of SOD2 in AD patients compared with NDAN and control subjects. A, B, The analyses conducted either on protein extracts from single individual synaptosomal fraction (A; ctrl vs AD, p = 0.0367; ctrl vs NDAN, p = 0.9987; AD vs NDAN, p = 0.0404) or on pooled extracts (B; ctrl vs AD, p = 0.0011; ctrl vs NDAN, p = 0.9988; AD vs NDAN, p = 0.0012) confirmed the preserved antioxidant content in NDAN individuals. Statistical analyses were made using one-way ANOVA (A: F(2,18) = 4.799, p = 0.0214; B: F(2,9) = 19.21, p = 0.0006) following Tukey's multiple-comparisons test. Values are expressed as fold change as a function of SYN ± SD (n = 7/group; 3 technical replicates). *p < 0.05; **p < 0.01. Download Figure 5-1, TIF file. [file ns-JN-RM-0295-20-s02.tif]

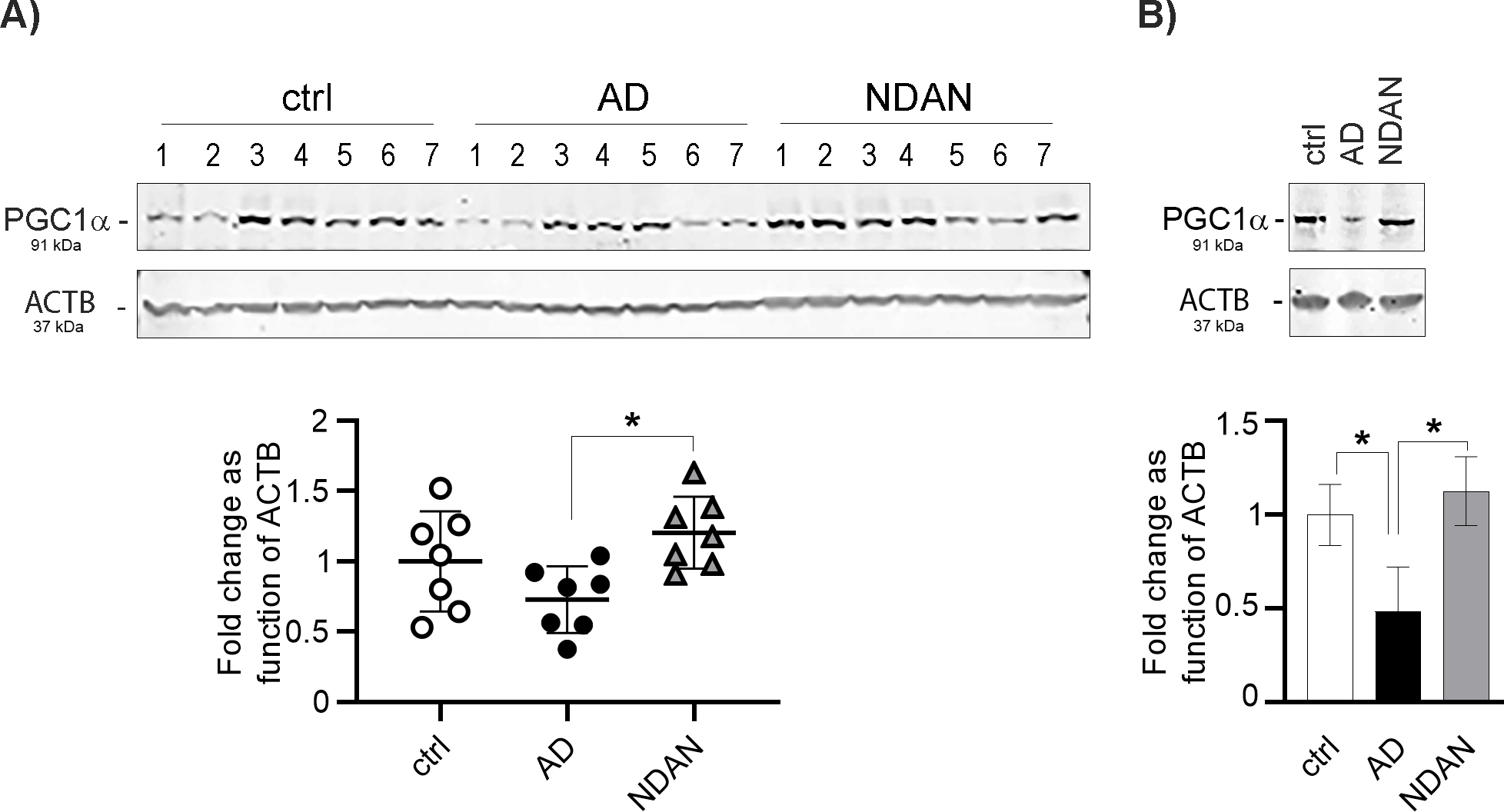

Supplement: Figure 6-1 — PGC1α expression in cortical total protein extracts. Wb analyses performed on total protein extracts showing a significant decrease of PGC1α in AD cortical samples compared with those from control and NDAN samples. A, B, The analyses conducted either on total protein extracts from single individuals (A: ctrl vs AD, p = 0.2094; ctrl vs NDAN, p = 0.3962; AD vs NDAN, p = 0.0163) or on pooled extracts (B: ctrl vs AD, p = 0.0426; ctrl vs NDAN, p = 0.7228; AD vs NDAN, p = 0.0169) indicate higher levels of PGC1α in NDAN subjects compared with AD patients. Statistical analyses were made using one-way ANOVA (A: F(2,18) = 4.829, p = 0.0209; B: F(2,6) = 8.924, p = 0.0159) following Tukey's multiple-comparisons test. Values are expressed as the fold change as function of ACTB ± SD (n = 7/group; 3 technical replicates). *p < 0.05. Download Figure 6-1, TIF file. [file ns-JN-RM-0295-20-s03.tif]

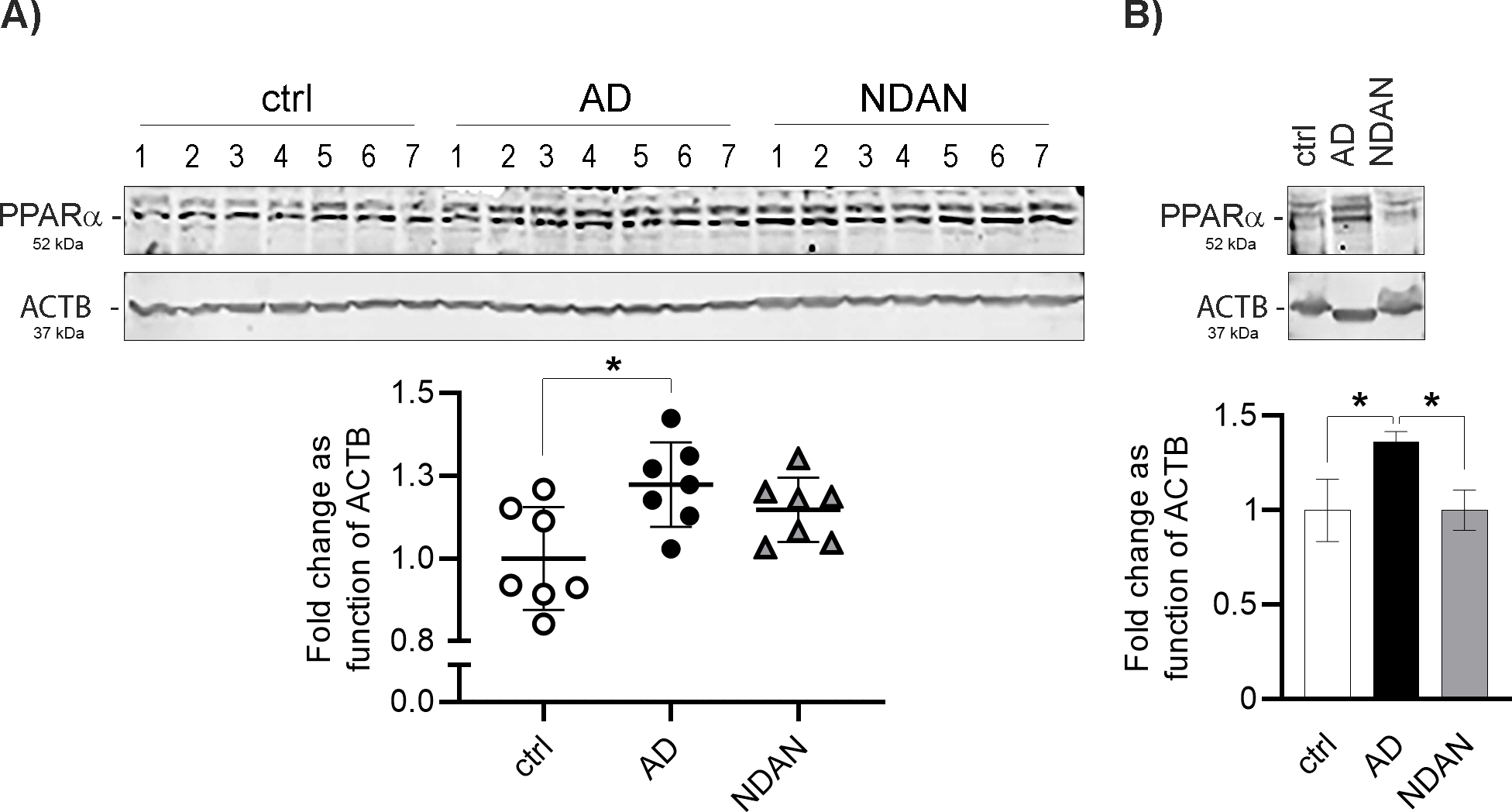

Supplement: Figure 7-1 — PPARα expression in cortical total protein extracts. Wb analyses performed on total protein extracts showing significant increase of PPARα in AD cortical samples compared with control and NDAN subjects. A, B, The analyses conducted either on total protein extracts from single individuals (A: ctrl vs AD, p = 0.0121; ctrl vs NDAN, p = 0.1106; AD vs NDAN, p = 0.5266) or on pooled total protein extracts (B: ctrl vs AD, p = 0.0223 ctrl vs NDAN, p = 0.9999; AD vs NDAN, p = 0.0227) indicate higher levels of PPARα in AD patients. Statistical analyses were made using one-way ANOVA (A: F(2,18) = 5.412, p = 0.0144; B: F(2,6) = 9.317, p = 0.0144) following Tukey's multiple-comparisons test. Values are expressed as the fold change as a function of ACTB ± SD (n = 7/group; 3 technical replicates). *p < 0.05. Download Figure 7-1, TIF file. [file ns-JN-RM-0295-20-s04.tif]

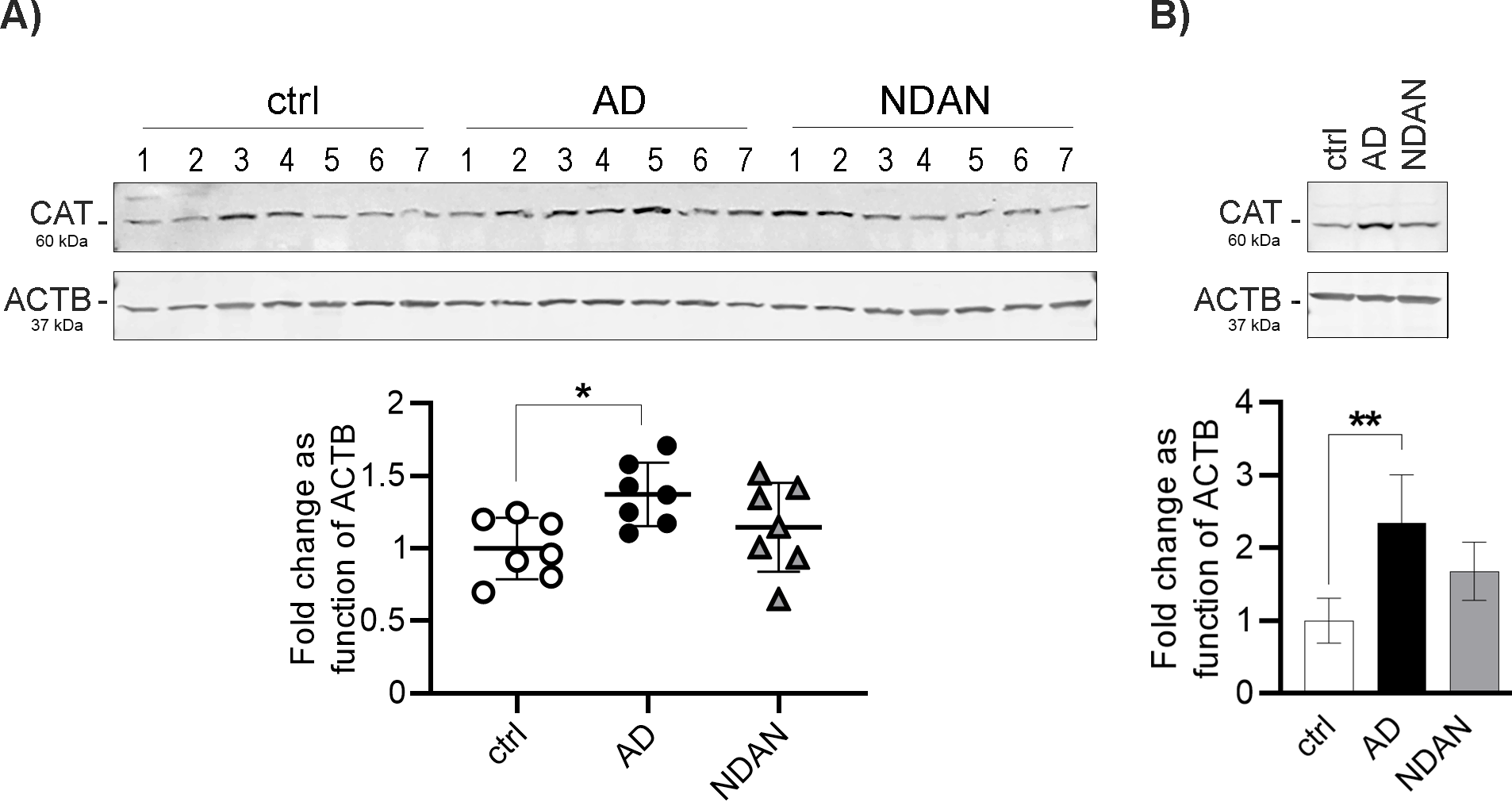

Supplement: Figure 8-1 — CAT expression levels in cytosolic fraction. Wb analyses performed on cytosolic fraction showing a significant increase of CAT in AD frontal cortices compared with those of control and NDAN subjects. A, B, Analyses conducted either on cytosolic protein extracts from single individuals (A: ctrl vs AD, p = 0.0297; ctrl vs NDAN, p = 0.5300; AD vs NDAN, p = 0.2265) or on pooled cytosolic protein extracts (B: ctrl vs AD, p = 0.0089; ctrl vs NDAN, p = 0.1711; AD vs NDAN, p = 0.1867) indicate higher levels of CAT in AD patients. Statistical analyses were made using one-way ANOVA (A: F(2,18) = 4.012, p = 0.0362; B: F(2,9) = 7.659, p = 0.0114) following Tukey's multiple-comparisons test. Values are expressed as the fold change as a function of ACTB ± SD (n = 7/group; 4 technical replicates). *p < 0.05; **p < 0.01. Download Figure 8-1, TIF file. [file ns-JN-RM-0295-20-s05.tif]
